# Systematic review of the Lancet Commission on Global Surgery indicators with quality assessment of modelled estimates

**DOI:** 10.1093/bjs/znaf289

**Published:** 2026-03-04

**Authors:** Theophilus T K Anyomih, Anita E Agbeko, Alazar B Aregawi, Kathryn Chu, Richard Crawford, Ewen M Harrison, Sivesh Kamarajah, Elizabeth Li, John G Meara, Albane Mulliez, Soha Sobhy, Richard Sullivan, Elizabeth Tissingh, Thomas G Weiser, Aneel Bhangu, Dmitri Nepogodiev

**Affiliations:** NIHR Global Health Research Unit on Global Surgery, University of Birmingham, Birmingham, UK; Ghana Hub, NIHR Global Health Research Unit on Global Surgery, University of Developmental Studies, Tamale, Ghana; Department of Surgery, School of Medical Sciences, Kwame Nkrumah University of Science and Technology, Kumasi, Ghana; College of Medicine and Health Sciences, Hawassa University, Hawassa, Ethiopia; Centre for Global Surgery, Stellenbosch University, Stellenbosch, South Africa; Department of Surgery, School of Clinical Medicine, Faculty of Health Sciences, University of the Witwatersrand, Johannesburg, South Africa; South African Hub, NIHR Global Health Research Unit on Global Surgery, University of the Witwatersrand, Johannesburg, South Africa; NIHR Global Health Research Unit on Global Surgery, University of Edinburgh, Edinburgh, UK; Centre for Medical Informatics, Usher Institute, University of Edinburgh, Edinburgh, UK; NIHR Global Health Research Unit on Global Surgery, University of Birmingham, Birmingham, UK; NIHR Global Health Research Unit on Global Surgery, University of Birmingham, Birmingham, UK; Program in Global Surgery and Social Change, Harvard Medical School, Boston, Massachusetts, USA; Department of Plastic and Oral Surgery, Boston Children’s Hospital, Boston, Massachusetts, USA; Medical School of Tours, Université de Tours (University of Tours), Tours, France; WHO Collaborating Centre for Global Women’s Health, University of Birmingham, Birmingham, UK; Institute of Cancer Policy, King’s College London, London, UK; King’s Global Health Partnerships, School of Life Course and Population Sciences, King’s College London, London, UK; Limb Reconstruction and Bone Infection Unit at the Royal National Orthopaedic Hospital Stanmore, London, UK; Department of Surgery, Stanford University, Palo Alto, California, USA; Wellcome Leap, San Diego, CA, USA; NIHR Global Health Research Unit on Global Surgery, University of Birmingham, Birmingham, UK; NIHR Global Health Research Unit on Global Surgery, University of Birmingham, Birmingham, UK

## Abstract

**Background:**

The Lancet Commission on Global Surgery (LCoGS) defined six indicators with 2030 targets to track national surgical system performance. The aim of this systematic review was to evaluate national reporting and attainment of benchmarks for each indicator and to assess the quality of modelling studies used to fill data gaps.

**Methods:**

Seven bibliographic databases (1 April 2015–24 July 2024) and government domains of 48 countries committed to National Surgical, Obstetric, and Anaesthesia Plans were searched. Records providing national estimates of any LCoGS indicator were eligible. The primary outcome was the proportion of World Bank-classified countries meeting indicator benchmarks and the secondary outcome was the quality of modelled national estimates. This systematic review was prospectively registered in PROSPERO, the international prospective register of systematic reviews (CRD420250650890).

**Results:**

Of 4245 records retrieved, 44 studies were included (35 research articles and 9 policy documents). Among 217 World Bank-classified countries, access to timely essential surgery (indicator 1) was reported for 94 countries (39% meeting benchmark), specialist surgical workforce density (indicator 2) was reported for 167 countries (50.3% meeting benchmark), surgical volume (indicator 3) was reported for 124 countries (31.5% meeting benchmark), perioperative mortality (indicator 4) was reported for 74 countries (no benchmark was set at country level), and financial risk protection indicators (indicators 5 and 6) were reported for five countries, with none meeting either benchmark. Across indicators, high-income countries were more likely to meet benchmarks. Most modelled studies lacked transparency in data sources, statistical methods, or model validation.

**Conclusion:**

Reporting of LCoGS indicators remains sparse and uneven, particularly in low- and middle-income countries. Without standardized, routine measurement and minimum quality standards for modelled estimates, progress towards 2030 cannot be credibly tracked. Integrating surgical metrics into national health information systems should be a policy priority.

## Teaser

This systematic review evaluated national reporting and benchmark attainment for all six Lancet Commission on Global Surgery indicators (2015–2024) across 217 World Bank-classified countries and assessed the methodological quality of modelled estimates used to fill data gaps. Reporting was sparse, particularly for financial risk protection (≤3% of countries), and benchmark achievement was strongly stratified by income, with no low-income country meeting workforce and surgical volume targets. Modelled estimates were frequently limited by poor transparency and lack of validation, underscoring the need for standardized reporting and integration of surgical metrics into national health information systems.

## Introduction

Strengthening surgical systems is recognized as a core component of universal health coverage (UHC) and health system resilience^1^. The 2015 Lancet Commission on Global Surgery (LCoGS) proposed six indicators: access to timely essential surgery (indicator 1), specialist surgical workforce density (indicator 2), surgical volume (indicator 3), perioperative mortality (indicator 4), financial risk protection against impoverishing expenditure (indicator 5), and financial risk protection against catastrophic expenditure (indicator 6)^2,3^. The Commission set targets for 2030 for each indicator to guide policy, financing, and research, drawing on learning from the WHO Safe Surgery Saves Lives programmes^4^. A subsequent consensus process merged the two financial indicators creating a streamlined framework of five indicators to improve transparency and utility^5^. Despite this, in this review, the two financial indicators are reported separately to retain comparability with previous LCoGS publications and the existing literature.

Despite global endorsement, and inclusion of four indicators (workforce, volume, and the two financial indicators) in the World Bank’s World Development Indicator (WDI) data set, national reporting remains inconsistent^6–8^. Most countries do not routinely publish these indicators and only a handful track them longitudinally^9^. This limits the ability to monitor progress toward achieving the LCoGS benchmarks^10^. These gaps are most marked in low-income countries (LICs) and lower middle-income countries (LMICs), where reliance on modelled estimates is common due to the absence of primary data^11,12^. Although modelling can fill information gaps, the quality and transparency of these estimates have not been systematically appraised.

Previous reviews have summarized indicator availability and achievement, but none has assessed both the completeness of reporting and the quality of modelled data at the national level^13–15^. Without robust, standardized, and repeatable reporting mechanisms, progress towards the LCoGS benchmarks cannot be reliably measured and surgical system strengthening risks being overlooked in UHC monitoring frameworks.

The aim of this study was to determine the extent of national reporting and benchmark attainment for each LCoGS indicator since 2015 and to evaluate the methodological quality of modelling studies used to generate national estimates.

## Methods

### Study design and reporting

This systematic review is reported according to the PRISMA 2020 guidelines^16^ and was prospectively registered in PROSPERO, the international prospective register of systematic reviews (CRD420250650890).

### Bibliographic database search

Seven databases were searched for English-language articles published from 1 April 2015 to 24 July 2024: MEDLINE, Embase (Ovid), Global Health (EBSCO), Science Citation Index (SCI) and Emerging Sources Citation Index (ESCI) via Web of Science, WHO Global Index Medicus, and Latin American and Caribbean Health Sciences Literature (LILACS). Search strategies combined controlled vocabulary (for example Medical Subject Heading (MeSH) terms for MEDLINE) and free-text terms for each indicator (*Table S1*). The database search was last updated on 17 March 2025; however, because the inclusion window ended on 24 July 2024, these 2025 updates did not identify any additional eligible records. Reference lists of included papers were screened and additional records were identified via expert consultation. Search results were deduplicated in EndNote and imported into Covidence for screening.

### Scoping search

To capture relevant policy documents commonly reported in government sources, a targeted web search was conducted on 24 June 2025. A Google Web search was conducted, restricted to government domains of 48 countries that have developed or committed to a National Surgical, Obstetric, and Anaesthesia Plan (NSOAP). Additionally, the health ministry websites of 13 of those countries with an NSOAP in active implementation were manually screened^17^. Structured queries combined indicator synonyms, policy document terms, and file type filters, while excluding academic domains. Details of search strings and domain blocks are available in *Supplementary Methods, Table S2, S3*. Search results were exported as csv, deduplicated in Excel, and screened.

### Eligibility criteria

Records were included if they reported on at least one LCoGS indicator at a national level and were published between 1 April 2015 and 24 July 2024. Reviews, non-English publications, subnational studies, and documents without numerical indicator data were excluded. Conference abstracts were not included due to lacking the details or methodological rigour necessary for reporting and decision-making. Unanalysed raw data sets were also not included as the time and specialist capacity required to process such data renders them an unrealistic basis for routine reporting by researchers or policymakers.

### Selection process

Titles/abstracts (or first pages) were screened independently by two reviewers (T.T.K.A. and A.M.). An initial calibration set of 100 records achieved ≥95% agreement; thereafter dual screening was applied to all remaining records. Full-text review followed the same procedure, with disagreements resolved by consensus or, if required, a third reviewer (D.N.).

### Data extraction and management

Data extraction was performed independently by two reviewers (T.T.K.A. and A.M.) using a standardized spreadsheet. Extracted fields included study metadata (author, country, and year), data level (national or multinational), indicator(s) reported, indicator values, data source/method (observed or modelled), and benchmark achievement status. For each indicator, results were stratified by World Bank income group^18^. Studies reporting modelled estimates were flagged for quality assessment. Extracted values were compared directly against the benchmark thresholds defined by the LCoGS to determine whether countries had achieved each target. *Table S4* outlines the indicators and corresponding benchmarks.

### Outcome definitions

The primary outcome was the proportion of World Bank-classified countries, stratified by income group, meeting the LCoGS targets.

For each indicator, the number and/or proportion of all 217 World Bank-classified countries^18^ that reported the indicator at least once, that achieved the benchmark, and that reported data more than once (serial reporting) were determined. For the purposes of this review, an indicator was considered ‘reported’ if any numerical value of the indicator was presented for a country, irrespective of whether the benchmark was met. An indicator was considered ‘achieved’ if the reported estimate met or exceeded the benchmark target defined by the LCoGS (*Table S4*). Where multiple data points were available for a country, the most recent estimate was used for the primary benchmark analysis. In instances where multiple data points were reported for the same time interval, selection was guided by a predefined hierarchy: prospective over retrospective design, observed over modelled data, and recency. Countries with two or more published estimates were classified as having serial reporting.

### Development of a quality assessment tool

Given the increasing reliance on modelled estimates in global surgical metrics, a structured evaluation of the methodological quality of such studies was warranted. To objectively evaluate the quality of studies reporting modelled estimates of LCoGS indicators and identify gaps for improvement, a quality assessment tool was developed based on the original 18-item Guidelines for Accurate and Transparent Health Estimates Reporting (GATHER) statement^19^, which was adapted to suit the indicators (*Table S5*). The GATHER statement outlines essential items for reporting modelled data, including disclosure of data sources, assumptions, and methods. Adherence to these principles enhances the interpretability and credibility of estimates, particularly when they are used to guide policy or investment decisions. The initial draft comprising 18 domains was grouped under four thematic sections: objectives, methodology, interpretation, and conflict of interest. Each item was scored on a three-point ordinal scale (low, intermediate, and high) based on predefined criteria reflecting completeness and transparency (*Table S6*).

### Expert consultation

The draft tool was refined via a structured online consultation with 15 international experts in surgical systems and health metrics, purposively selected to ensure representation across diverse geographical regions and income settings. Each participant rated the importance of the 18 proposed domains using a five-point Likert scale (1 = not important to 5 = very important). Domains were retained if ≥60% of respondents rated them as four or five. Participants were also invited to provide qualitative feedback on wording, clarity, and interpretation, which was used to inform the final version of the tool. Participation was voluntary and anonymous, and the consultation was conducted in a single round. This approach enabled the identification of key domains deemed essential by the expert panel and informed the final version of the assessment tool.

### Data analysis

Descriptive statistics are used to summarize the number and proportion of all 217 World Bank-classified countries^18^ reporting and achieving each LCoGS indicator benchmark. Results are stratified by income group. Meta-analysis was not undertaken due to substantial heterogeneity in data sources, study design, and reporting time frames across included studies.

## Results

### Study selection and characteristics

**Fig. 1:**
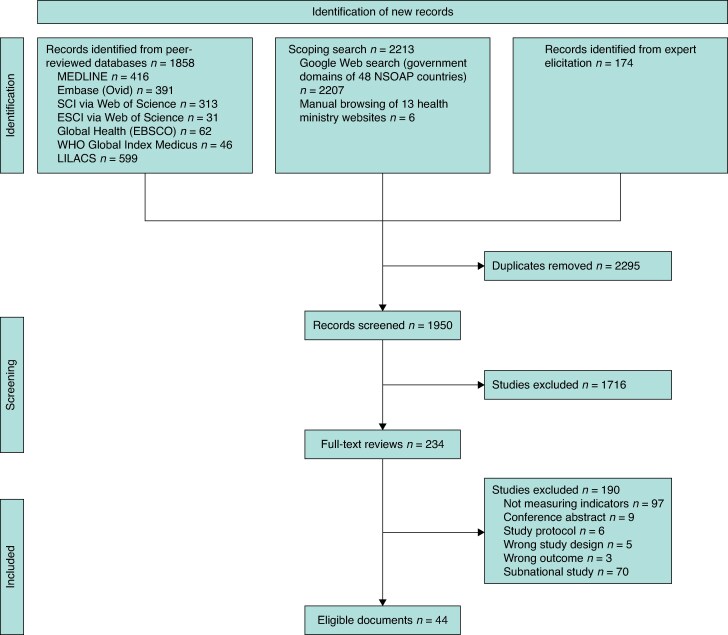
PRISMA flow diagram summarizing study selection, including the number of records identified, screened, assessed for eligibility, and included in the final systematic review SCI, Science Citation Index; ESCI, Emerging Sources Citation Index; LILACS, Latin American and Caribbean Health Sciences Literature; NSOAP, National Surgical, Obstetric, and Anaesthesia Plan.

Comprehensive searches retrieved 4245 records (1858 from bibliographic databases, 2213 from the scoping search, and 174 through expert elicitation; *Fig. 1*). After removal of 2295 duplicates, 1950 records were screened, 234 underwent full-text review, and 44 records met the inclusion criteria: 35 peer-reviewed studies^10,20–53^ and 9 government policy documents^54–62^. Of the 44 included studies, 29 were national-level studies^20–24,26–30,33,35,37,38,40,43,45–47,51,53–62^, 6 used prospective data^20,25,31,38,41,44^, and 17 relied on modelled estimates for at least one indicator^10,22,28,32,34,35,39,40,42–46,48–50,52^. Only five studies^27,33,43–45^ reported on all six indicators (*Table 1* and *Table 2*).

**Table 1:** Summary of included peer-reviewed studies mapped against the LCoGS indicators

Study	Year	Study type	Level	Country/region	Income	Data	Indicators
Pérez Rivera *et al*.^20^	2024	Prospective	National	Colombia	UMIC	Observed	4
Hellar *et al*.^63^	2024	Retrospective	National	Tanzania	LMIC	Observed	2
Pérez-Soto *et al*.^22^	2023	Retrospective	National	Mexico	UMIC	Modelled	1–3
Zadey^23^	2023	Retrospective	National	India	LMIC	Observed	3
Cook *et al*.^24^	2024	Retrospective	National	Ethiopia	LIC	Observed	3 and 4
Qin *et al*.^25^	2023	Prospective	Multinational	Asia/Pacific*	UMIC and LMIC	Observed	2–4
Adde *et al*.^51^	2023	Retrospective	National	Liberia	LIC	Observed	1
Lim *et al*.^53^	2023	Retrospective	National	Philippines	LMIC	Observed	1
Samper *et al*.^26^	2022	Retrospective	National	Colombia	UMIC	Observed	3 and 4
Nunez *et al*.^27^	2022	Retrospective	National	Mongolia	UMIC	Observed	1–6
Buda *et al*.^28^	2022	Retrospective	National	Guatemala	UMIC	Modelled	1
Hoh *et al*.^29^	2021	Retrospective	National	Malaysia	UMIC	Observed	1
Odinkemelu *et al*.^30^	2021	Retrospective	National	Liberia	LIC	Observed	2 and 3
Pouramin *et al*.^31^	2020	Prospective	Multinational	Multiregional*	LMIC and LIC	Observed	1
Nagra *et al*.^32^	2020	Retrospective	Multinational	Pacific*	All	Modelled	3 and 4
Hanna *et al*.^33^	2020	Retrospective	National	Colombia	UMIC	Observed	1–4
Wigley *et al*.^34^	2020	Retrospective	Multinational	Sub-Saharan Africa*	All	Modelled	1
Gyedu *et al*.^35^	2020	Retrospective	National	Ghana	LMIC	Modelled	3
Bouchard *et al*.^36^	2020	Retrospective	Multinational	Multiregional*	LIC	Observed	2
Dahir *et al*.^37^	2020	Retrospective	National	Somaliland	LIC	Observed	1–3, 5, and 6
van Duinen *et al*.^38^	2020	Prospective	National	Sierra Leone	LIC	Observed	1
Holmer *et al*.^10^	2019	Retrospective	Multinational	Multiregional*	All	Modelled	1–4
Juran *et al*.^52^	2018	Retrospective	Multinational	Sub-Saharan Africa	LIC, LMIC, and UMIC	Modelled	1
Ouma *et al*.^39^	2018	Retrospective	Multinational	Sub-Saharan Africa*	LIC, LMIC, and UMIC	Modelled	1
Gyedu *et al*.^40^	2018	Retrospective	National	Ghana	LMIC	Modelled	3
Biccard *et al*.^41^	2018	Prospective	Multinational	Sub-Saharan Africa*	LMIC	Observed	2–4
Knowlton *et al*.^42^	2017	Retrospective	Multinational	Multiregional*	LIC and LMIC	Modelled	1
Massenburg *et al*.^43^	2017	Retrospective	National	Brazil	UMIC	Modelled	1–6
Guest *et al*.^44^	2017	Prospective	Multinational	Pacific*	All	Modelled	1–4
Bruno *et al*.^45^	2017	Retrospective	National	Madagascar	LIC	Modelled	1–6
Stewart *et al*.^46^	2016	Retrospective	National	Ghana	LMIC	Modelled	1
Esquivel *et al*.^47^	2016	Retrospective	National	Zambia	LIC	Observed	1
Weiser *et al*.^47^	2016	Retrospective	Multinational	Multiregional*	All	Modelled	3
Alkire *et al*.^49^	2015	Retrospective	Multinational	Multiregional*	All	Modelled	1
Holmer *et al*.^50^	2015	Retrospective	Multinational	Multiregional*	All	Modelled	2

^*^Region. LCoGS, Lancet Commission on Global Surgery; UMIC, upper middle-income country; LMIC, lower middle-income country; LIC, low-income country.

**Table 2:** Summary of included policy documents

Document title	Year	Level	Country/region*	Income	Indicators
NSOAP for Ghana^61^	2024	National	Ghana	LMIC	1–3
NSOAP for Namibia^62^	2023	National	Namibia	UMIC	2 and 3
National Vision for Surgical Care 2020–2025^57^	2021	National	Pakistan	LMIC	2
Action Framework for Safe and Affordable Surgery in the Western Pacific Region (2021–2030)^60^	2021	Multinational	Pacific*	LMIC and LIC	1
NSOANP for Nigeria^54^	2019	National	Nigeria	LMIC	1–3, 5, and 6
NSOAP for Tanzania^58^	2018	National	Tanzania	LIC	2 and 3
NSOAP for Rwanda^56^	2018	National	Rwanda	LIC	1–4
NSOASP for Zambia^55^	2017	National	Zambia	LMIC	2 and 3
SaLTS Strategic Plan 2016–2020^59^	2016	National	Ethiopia	LIC	2 and 3

^*^Region. NSOAP, National Surgical, Obstetric, and Anaesthesia Plan; LMIC, lower middle-income country; UMIC, upper middle-income country; LIC, low-income country; NSOANP, National Surgical, Obstetrics, Anaesthesia, and Nursing Plan; NSOASP, National Surgical, Obstetric, and Anaesthesia Strategic Plan; SaLTS, Saving Lives Through Safe Surgery.

### Indicator reporting and benchmark attainment

Access to timely essential surgery (indicator 1) was reported by 94 countries (17 high-income countries (HICs), 19 upper middle-income countries (UMICs), 36 LMICs, and 22 LICs) representing 43.3% (94 of 217) of all World Bank-classified countries. Benchmark achievement (≥80% population coverage within 2 h) was highest in HICs (94% (16 of 17)), followed by UMICs (47% (9 of 19)), LMICs (22% (8 of 36)), and LICs (18% (4 of 22)). Serial reporting was identified in 51 countries (8 UMICs, 20 LMICs, 23 LICs, and 0 HICs).

Specialist surgical workforce density (indicator 2) was the most reported indicator with data from 167 countries (60 HICs, 47 UMICs, 41 LMICs, and 19 LICs) representing 77.0% (167 of 217) of all countries. No LICs met the workforce target of ≥20 surgical, anaesthetic, and obstetric providers per 100 000 population. The proportion meeting the benchmark was highest in HICs (90% (54 of 60)), followed by UMICs (51% (24 of 47)) and LMICs (15% (6 of 41)). Only eight countries provided more than one estimate over time.

Surgical volume (indicator 3) was reported by 124 countries (43 HICs, 31 UMICs, 31 LMICs, and 19 LICs) covering 57.1% (124 of 217) of all countries. No LMICs or LICs met the target of ≥5000 procedures per 100 000 population. The benchmark was met by most HICs (77% (33 of 43)) and a few UMICs (19% (6 of 31)). Repeated reporting was observed in 11 countries.

Perioperative mortality (indicator 4) was reported at least once by 74 countries (17 HICs, 23 UMICs, 19 LMICs, and 15 LICs) representing 34.1% (74 of 217) of all countries. No benchmark was set at country level. The commission set a global target for universal tracking at a national level by all countries.

**Fig. 2:**
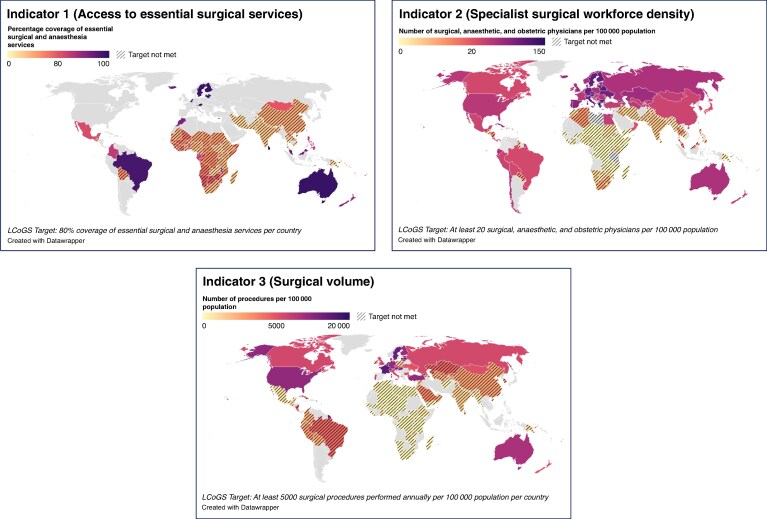
World maps showing country-level attainment of LCoGS benchmarks **a** For indicator one (access to timely essential surgery). **b** For indicator two (specialist surgical workforce density). **c** For indicator three (surgical volume). Countries are classified as meeting the benchmark, not meeting the benchmark, or having no available data. Indicators four–six are not shown. Indicator four (perioperative mortality) was excluded as the LCoGS target applies at a global level rather than to individual countries. Indicators five and six (financial risk protection) were excluded due to limited reporting (only five countries had available data) precluding meaningful global visualization. LCoGS, Lancet Commission on Global Surgery.

Financial risk protection indicators (indicators 5 and 6) were the least reported, with only 2.3% (5 of 217) of countries providing any data. Of these, two were UMICs, one was a LMIC, and two were LICs. No HICs reported data on either indicator. None of the five countries met the targets of 100% financial risk protection against impoverishing expenditure and 100% financial risk protection against catastrophic expenditure (see *Fig. 2* and *Table 3*).

**Table 3:** Proportion of countries meeting LCoGS indicator benchmarks, by country income group

Indicator	Target	Definitions	HIC	UMIC	LMIC	LIC	Total
Access to timely essential surgery	A minimum of 80% coverage of essential surgical and anaesthesia services per country by 2030	Reporting*	20% (17 of 86)	35% (19 of 54)	71% (36 of 51)	85% (22 of 26)	43.3% (94 of 217)
		Benchmark met†	94% (16 of 17)	47% (9 of 19)	22% (8 of 36)	18% (4 of 22)	39% (37 of 94)
		Serial reporting‡	0	8	20	23	
Specialist surgical workforce density	100% of countries with at least 20 surgical, anaesthetic, and obstetric physicians per 100 000 population by 2030	Reporting*	70% (60 of 86)	87% (47 of 54)	80% (41 of 51)	73% (19 of 26)	77.0% (167 of 217)
		Benchmark met†	90% (54 of 60)	51% (24 of 47)	15% (6 of 41)	0% (0 of 19)	50.3% (84 of 167)
		Serial reporting‡	2	3	2	1	
Surgical volume§	80% of countries by 2020 and 100% of countries by 2030 tracking surgical volume	Reporting*	50% (43 of 86)	57% (31 of 54)	61% (31 of 51)	73% (19 of 26)	57.1% (124 of 217)
	5000 procedures per 100 000 population by 2030	Benchmark met†	77% (33 of 43)	19% (6 of 31)	0% (0 of 31)	0% (0 of 19)	31.5% (39/124)
		Serial reporting‡	0	6	3	2	
Perioperative mortality§#	80% of countries by 2020 and 100% of countries by 2030 tracking perioperative mortality	Reporting*	20% (17 of 86)	43% (23 of 54)	37% (19 of 51)	58% (15 of 26)	34.1% (74/217)
	In 2020, assess global data and set national targets for 2030		Not reported				
Financial risk protection against impoverishing expenditure	100% protection against impoverishment from out-of-pocket payments for surgical and anaesthesia care by 2030	Reporting*	0% (0 of 86)	4% (2 of 54)	2% (1 of 51)	8% (2 of 26)	2.3% (5 of 217)
		Benchmark met†	Not reported	0% (0 of 2)†	0% (0 of 1)	0% (0 of 2)	0
		Serial reporting‡	Not reported	0	0	0	
Financial risk protection against catastrophic expenditure	100% protection against catastrophic expenditure from out-of-pocket payments for surgical and anaesthesia care by 2030	Reporting*	0% (0/86)	4% (2 of 54)	2% (1 of 51)	8% (2 of 26)	2.3% (5 of 217)
		Benchmark met†	Not reported	0% (0 of 2)†	0% (0 of 1)	0% (0 of 2)	0
		Serial reporting‡	Not reported	0	0	0	

^*^Denominators are based on the number of countries in that income group. †Denominators are based on the number of countries with available data. ‡Number of countries with indicator reported two or more times. §Indicators with two targets set by the LCoGS. #The LCoGS set a target for universal national reporting of perioperative mortality but did not define a specific numerical mortality rate benchmark. Therefore, ‘Benchmark met’ is not calculated for this indicator. LCoGS, Lancet Commission on Global Surgery; HIC, high-income country; UMIC, upper middle-income country; LMIC, lower middle-income country; LIC, low-income country.

### Quality of modelling studies

**Fig. 3:**
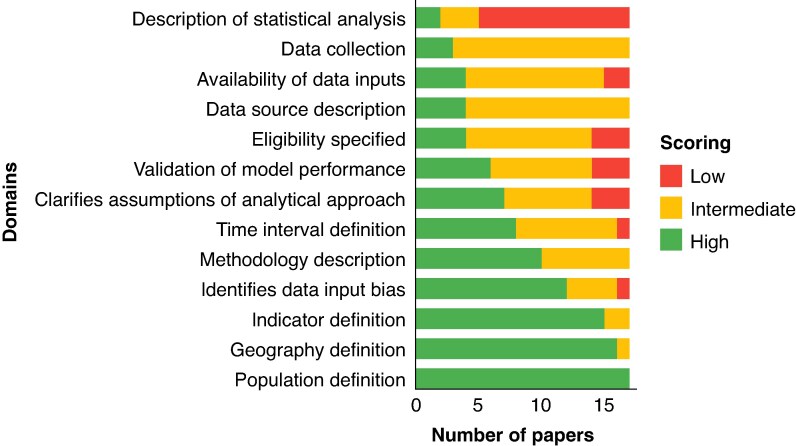
Domain-level quality ratings (for domain 1 to domain 13) for the modelled studies included in the review Bars show the proportion of studies scoring high, intermediate, or low quality for each domain according to the final quality assessment tool.

After the expert consultation process, five domains (7, 8, 14, 15, and 18) were excluded based on failing to meet the predefined threshold of ≥60% of experts rating them as ‘important’ or ‘very important’. The final version of the quality assessment tool included 13 domains, grouped into three thematic sections, and is summarized in *Table 4*. Seventeen studies^10,22,28,32,34,35,39,40,42–46,48–50,52^ were assessed for quality using the final tool. Four studies had at least 8 of 13 domains rated as high quality. Performance across domains was variable. Objectives and context of modelling were generally well described, with most studies scoring ‘high’ for clearly defining the indicator (domain 1) (16 of 17 studies (94%)), target population (domain 2) (17 of 17 studies (100%)), and geographical scope (domain 3) (16 of 17 studies (94%)). Definitions of the time interval (domain 4) (8 of 17 studies (47%)) and modelling methodology (domain 5) (9 of 17 studies (53%)) were more inconsistently reported. Data sources and transparency were generally weak. Only 18% of studies (3 of 17) described prospective input data sources (domain 6). Inclusion and exclusion criteria, data source characteristics, and metadata availability (domains 7–9) were each rated ‘high’ in only 4 of 17 (24%) studies. Just 6 of 17 studies (35%) described all steps in model construction (domain 10) and only 2 of 17 studies (12%) performed any form of validation against observed data (domain 11). Potential biases (domain 12) were explicitly discussed in 11 of 17 studies (65%), while assumptions (domain 13) were clearly identified and explored in 7 of 17 studies (41%). Overall, while reporting on scope and intent was relatively strong, method transparency and quality assurance measures were frequently lacking (*Fig. 3*).

**Table 4:** Quality assessment tool for modelled estimates of LCoGS indicators (based on adapted GATHER statement^19^)

Domain	Quality		
	Low	Intermediate	High
**Objectives**			
1. Provide a clear definition of the Lancet Commission indicator(s) used	No explicit definition	Definition used is slightly different to the ones used in the Lancet Commission indicators	Provides a clear definition of the indicators used, similar to the Lancet Commission indicators
2. Provide a clear definition of the target population for which the modelling analysis is applicable to	No description of the population	There is a vague definition of the population (that is age or sex) or patients included in the study	Clear definition including what procedures are included and excluded
3. Clear definition of the geography studied	No definitions of the scale of country or regions included	Clear definition of region covered but have had to model some of the data or have incomplete data for the region	Clearly defined catchment (for example province or state-level or national-level analysis) with complete population data
4. Clear definition of the time interval	Not defined	Poorly defined	Clearly defined
**Methodology**			
5. Description of methodology	Minimal or no description of methodology	Clearly described in text	Clearly described with conceptual overview (for example flow chart or diagram) and including mathematical formulae/statistical code if applicable
6. Methodology of how underlying data were collected	No description of the nature of data collection for the inputs reported in the study	Data inputs obtained were retrospective in nature including administrative data, with high risk of potential biases	Data inputs were prospectively collected, either from cohort studies or RCTs
7. Specify the inclusion and exclusion criteria	No clear specification around inclusion or exclusion criteria for the data source(s) being used	Partial specification around inclusion or exclusion criteria for the data source(s) being used	Full specification around inclusion or exclusion criteria for the data source(s) being used
8. Description of data source characteristics	No information on data source or characteristics	Provides information on all included data sources and their main characteristics.	Provides information on all included data sources and their main characteristics and identifies and describes any categories of input data that have potentially important biases
9. Availability of data inputs	Not available	Data sources clearly referenced but data not provided or contacts of rights to data not provided	Provides all data inputs (including all relevant meta-data) in an extractable file format; make these available as a supplementary file, or via a clearly identified contact/data custodian who retains the rights to the data
10. Description of statistical analysis	Minimal or no description of statistical analysis	Clearly described in text but no statistical or mathematical code provided or how they can be assessed	Detailed description of all steps of the analysis (data cleaning, data preprocessing, data adjustments, and weighting of data sources) including mathematical formulae; mathematical or statistical model(s) available in the supplementary file
11. Validation of model performance	No attempt to validate the model	Attempt to validate the underlying methodology	Attempt to validate modelled data against observed data and detailed description of how validation was done
**Interpretation**			
12. Identification of potential biases relating to data inputs	Not performed	Biases identified but their potential impact on findings not discussed	Biases identified and their potential impact on findings discussed
13. Identification of assumptions within analytical approach	Not performed	Assumptions identified but their potential impact on findings not discussed	Assumptions identified and their potential impact on findings discussed

LCoGS, Lancet Commission on Global Surgery; GATHER, Guidelines for Accurate and Transparent Health Estimates Reporting.

## Discussion

Across 217 World Bank-classified countries, fewer than half have ever published an estimate for timely access to essential surgery, only one-third have reported a perioperative mortality rate, and financial risk protection has only been documented in five countries. Benchmark attainment mirrors this data gap: almost all HICs meet the access target, yet no LIC reaches the workforce and surgical volume benchmarks. Repeated reporting is rare, limiting the ability to monitor change over time. Although modelling studies attempt to bridge these gaps, their transparency, reproducibility, and methodological rigour are often suboptimal.

Despite endorsement by global bodies and inclusion in the WDI data set in 2016, reporting of LCoGS indicators fell in 2023^6,7^. This has been attributed to barriers such as underdeveloped data infrastructure, fragmented care delivery, and lack of investment, particularly in LMICs/LICs^64^  ^,65^. Within countries, weak multisectoral coordination and variable case mix further inhibit the integration of these indicators into routine health information systems^11^. By contrast, other domains of global health, such as maternal and neonatal health and infectious disease programmes, use standardized indicator frameworks that facilitate consistent tracking and measurable gains^66–68^. Of all six indicators, indicator one (access to timely essential surgery) is among the most difficult to assess globally. In high-income settings, near-universal hospital coverage means travel times are routinely below 2 h but are rarely reported as surgical metrics. Instead, access data are embedded within broader infrastructure or emergency-care statistics. Consequently, reporting for this indicator is concentrated in LMICs, where modelling is often used to quantify deficits, while in HICs its absence reflects reporting priorities. Although HICs are generally assumed to have adequate geographical coverage, this does not preclude substantial subnational inequities in access for different population groups^69–72^. There is a need for HICs to generate and publish granular data on access to essential surgical care. Strengthening frameworks for data collection in global surgery will enhance reporting, enable benchmarking of progress, and identify areas requiring intervention.

Benchmark achievement was highly variable and mirrored human development index levels. While nearly all HICs met the surgical workforce density target, none of the LICs achieved this benchmark. In addition, the surgical volume benchmark was not met by any LMIC or LIC. Financial risk protection indicators were the least reported and none of the five countries with available data met the 100% coverage target. These findings are consistent with previous estimates and underscore both the lack of data systems and the absence of formal mechanisms to support indicator-based benchmarking in global surgery^3,10,73^. Without reliable denominators and repeated measurement, benchmarking becomes a theoretical exercise rather than a tool for accountability. The availability of disaggregated, country-level data has also supported subnational accountability and enabled targeted improvements. Within surgery for instance, national-level audit programmes such as the UK’s National Emergency Laparotomy Audit (NELA), Dutch Institute for Clinical Auditing (DICA), and the US National Surgical Quality Improvement Program (NSQIP) have demonstrated that structured indicator tracking can improve outcomes^74–77^. NELA has been associated with reductions in postoperative mortality and has enhanced accountability by providing regular feedback to hospitals and clinicians^78^. In contrast, the global surgical field lacks an equivalent mechanism for standardized indicator tracking, which undermines accountability and inhibits system-wide improvement.

In the absence of primary data, modelled estimates have gained prominence^13–15^. Yet, as this review shows, the quality of modelling studies varies considerably. Of the 16 studies assessed, only 4 met high-quality criteria in ≥8 of 13 domains. While the majority clearly defined their objectives and target populations, most studies failed to report data inputs transparently or describe their analytical methods in sufficient detail. Less than a third acknowledged assumptions or uncertainty, and only 25% validated their models against observed data. These deficiencies limit the interpretability and applicability of modelling outputs for national planning or international comparison.

This review addresses an important evidence gap by consolidating national-level reporting across all six LCoGS indicators worldwide and screening of government domains of 48 NSOAP countries while applying a novel quality appraisal tool for modelling studies. The protocol was prospectively registered and duplicate screening and extraction ensured methodological rigour. However, there were some limitations. First, although data from non-published sources (including governmental reports and policy documents) was searched for and incorporated, pre-prints, conference abstracts, and unanalysed databases, which may contain relevant data, were excluded. Second, the scoping search was confined to government domains of 48 countries committed to NSOAP development, potentially overlooking records from non-NSOAP countries. Some national surgical plans and related policy documents may exist but are not publicly available or may have limited discoverability through open-source searches. For example, Ecuador’s National Surgical Strategic Plan (NSSP) is known to exist but is not readily accessible^79^. This highlights a need for health ministries to ensure national reporting systems are transparent and publicly available. Third, the academic literature is largely skewed towards LMICs and may under-represent reporting from HICs, where data are often collected and stored through systems such as the Organisation for Economic Co-operation and Development (OECD), Demographic and Health Surveys (DHS), or the Healthcare Cost and Utilization Project (HCUP)^80–82^, which were outside the remit of this review. Fourth, heterogeneity in data sources, time frames, and health system structures limited direct comparisons across countries and reliable cross-national typologies for financing and expenditure remain uneven. Fifth, reporting remains sparse, especially in LICs, leading to potential under-representation of some settings. Finally, the restriction to English-language publications may have excluded evidence from non-English-speaking regions. This reflects a broader structural bias in global health research, where English-language dominance can obscure substantial data published in other major languages. To improve equity and completeness, future reviews should include multilingual search strategies or follow-up studies in major languages to make global surgery evidence truly global. Future studies could examine how indicator reporting and achievement vary by health system model and national health expenditure. Mapping countries by financing type and spending levels could reveal which system structures most support robust surgical metrics and financial risk protection.

Surgical indicators remain disconnected from institutionalized reporting infrastructure that gives other global health fields visibility and therefore priority. Without routine collection and reporting, surgical need is excluded from UHC benefit packages, official development assistance (ODA) allocations, and sustainable development goal (SDG) progress reviews. This invisibility perpetuates a cycle of marginalization, limiting both domestic investment and external financing for surgical care.

By contrast, maternal and newborn health programmes have advanced through coordinated indicator frameworks, dedicated financing, and regularized data collection using platforms such as DHS and Multiple Indicator Cluster Surveys (MICS). These models are transferable. Surgery does not require a new system from scratch but rather integration into proven mechanisms. The challenge is mainly structural: the absence of a unified framework continues to undermine policy action and resource allocation in this complex, high-cost area.

Embedding surgical indicators into national health information systems is essential. Existing platforms such as District Health Information Software 2 (DHIS2)—used by over 80 countries—and the WHO Operative Encounter Registry (OER) offer practical infrastructure for integrating surgical metrics^83,84^. These should be aligned with global standards on indicator definitions, thresholds, and reporting cycles. WHO must provide normative leadership by standardizing indicators and establishing minimum reporting cycles, while multilateral development banks (MDBs) should provide financing on the condition of reliable reporting. Bilateral funders should support data system investments that enable surgical metrics to be tracked alongside UHC indicators. These reforms would close visibility gaps, enable LMIC-led priority setting, and foster equity, transparency, and accountability in surgical system strengthening.

## Supplementary Material

znaf289_Supplementary_Data

## Data Availability

All data used in this study were extracted from previously published sources. No new primary data were generated. Extracted data and the analytical code are available from the corresponding author upon reasonable request.
