# Supplementary material for: Systematic review of the Lancet Commission on Global Surgery indicators with quality assessment of modelled estimates
Source: Br J Surg. 2026 Mar 4;113(3):znaf289. doi: 10.1093/bjs/znaf289 (PMC13016785; doi:10.1093/bjs/znaf289)
Supplement: znaf289_Supplementary_Data [file znaf289_supplementary_data.docx]

**Systematic review of the Lancet Commission on Global Surgery (LCoGS) indicators with quality assessment of modelled estimates**

Theophilus T.K. Anyomih¹, Anita E. Agbeko²˒³, Alazar B. Aregawi⁴, Kathryn Chu⁵, Richard Crawford⁶˒⁷, Ewen M. Harrison⁸˒⁹, Sivesh Kamarajah¹, Elizabeth Li¹, John G. Meara¹⁰˒¹¹, Albane Mulliez¹², Soha Sobhy MD¹³, Richard Sullivan¹⁴, Elizabeth Tissingh¹⁵˒¹⁶, Thomas G. Weiser¹⁷˒¹⁸, Aneel Bhangu¹, and Dmitri Nepogodiev¹

^1^ NIHR Global Health Research Unit on Global Surgery, University of Birmingham, UK

^2^ Ghana Hub, NIHR Global Health Research Unit on Global Surgery, University of Developmental Studies, Tamale, Ghana

^3^ Kwame Nkrumah University of Science and Technology, Kumasi, Ghana

^4^ College of Medicine and Health Sciences, Hawassa University, Ethiopia

^5^ Centre for Global Surgery, Stellenbosch University, South Africa

^6^ Department of Surgery, School of Clinical Medicine, Faculty of Health Sciences, University of the Witwatersrand, Johannesburg, South Africa

^7^ South African Hub, NIHR Global Health Research Unit on Global Surgery, University of the Witwatersrand, Johannesburg, South Africa

^8^ NIHR Global Health Research Unit on Global Surgery, University of Edinburgh, UK

^9^ Centre for Medical Informatics, Usher Institute, University of Edinburgh, UK

^10^ Program in Global Surgery and Social Change, Harvard Medical School, Boston, USA

^11^ Department of Plastic and Oral Surgery, Boston Children’s Hospital, Boston, USA

^12^ Université de Tours (University of Tours, France)

^13^ WHO Collaborating Centre for Global Women’s Health, University of Birmingham, UK

^14^ Institute of Cancer Policy, King’s College London, UK

^15^ King’s Global Health Partnerships, King’s College London, UK

^16^ Royal National Orthopaedic Hospital, London, UK

^17^ Department of Surgery, Stanford University, Palo Alto, USA

^18^ Wellcome Leap

**Corresponding author.** Dr Theophilus TK Anyomih, NIHR Global Health Research Unit on Global Surgery, Institute of Applied Health Research, University of Birmingham, Birmingham B15 2TH, UK ([t.t.k.anyomih@bham.ac.uk](mailto:t.t.k.anyomih@bham.ac.uk))

**ORCID ID:** <https://orcid.org/0000-0001-6684-5821>

**Twitter: @theophilusteddy**

**LinkedIn:** <https://www.linkedin.com/in/theophilusteddy/>

**Supplementary Materials - Index**

| **Supplementary Methods** |  |
| --- | --- |
| *Details of scoping search*  *Policy block*  *File-type & exclusion block* | *page 3* |
| **Supplementary Results** |  |
| Overview | *page 5* |
| **Supplementary Appendixes** |  |
| PRISMA 2020 Checklist | *page 6* |
| **Supplementary Figures and Tables** |  |
| Table S1. Bibliographic database search strategy for identification of peer-reviewed studies | *page 8* |
| Table S2. Search terms for each Lancet Commission on Global Surgery (LCoGS) indicator (Indicator blocks) | *page 9* |
| Table S3. Country domain blocks used for Google web search (site: operators) | *page 10* |
| Table S4. Definitions and global targets for the six Lancet Commission on Global Surgery indicators | *page 11* |
| Table S5. GATHER (Guidelines for Accurate and Transparent Health Estimates Reporting) checklist items | *page 12* |
| Table S6. Initial draft of the modelling study quality-assessment tool (pre-consultation version) | *page 14* |
| Table S7. Countries represented in the review by World Bank income group | *page 16* |
| Table S8. Total World Bank countries by income group (2025 classification) | *page 16* |
| Tables S9. Country-level values and benchmark status for indicators 1 (Access) | *page 17* |
| Tables S10. Country-level values and benchmark status for indicators 2 (Workforce) | *page 20* |
| Tables S11. Country-level values and benchmark status for indicators 3 (Volume) | *page 24* |
|  |  |
| **References** | *page 28* |
|  |  |

**Supplementary Methods**

**Scoping search**

The full strategy for the bibliographic database search is shown in Supplementary Table 1. To capture non-indexed national policy documents reporting one or more Lancet Commission on Global Surgery (LCoGS) indicators, we conducted a structured Google Web Search targeting official government domains for 48 countries with active or planned National Surgical, Obstetric and Anaesthesia Plans (NSOAPs). Search syntax combined three concept blocks:

1. **Indicator block** – phrases specific to each of the six LCoGS indicators (e.g., *“surgical volume 5000”*, *“SAO density”*, *“perioperative mortality rate”*) (Supplementary Table 2);
2. **Policy block** – terms identifying strategic, governmental, or situational reports (e.g., *“policy brief”*, *“national surgical plan”*, *NSOAP*, *“health sector plan”*) (Supplementary Table 3);
3. **Domain block** – site: operators restricted to official government domains (Supplementary Table 3).

Because Google limits queries to 32 site: operators, the country list was split into three mutually exclusive “site-blocks” (A, B, C). Each indicator was searched separately across all three blocks to achieve complete coverage. File-type filters *(filetype:(pdf OR doc OR docx*)) were applied to prioritise document-based outputs, and an exclusion block removed bibliographic databases, journal platforms, and generic academic content (e.g., *-site:ovid.com, -site:scopus.com, -"journal article").*

Searches were run in an incognito Chrome browser (results/page = 100; SafeSearch = off) with a custom date range from 1 January 2019 to the search date (24 June 2025). For each query, the first five pages of results (≤500 hits) were exported as csv, deduplicated in Excel, and tagged by indicator, site-block, and harvest date. All included URLs were screened independently by two reviewers, with disagreements resolved by consensus. Documents meeting the inclusion criteria (government or multilateral source, indicator data, national scope, English language) were retained for data extraction.

**Policy block**

("policy brief" OR "national surgical plan" OR NSOAP OR strategy OR roadmap OR "white paper" OR "country report" OR "situation analysis" OR "health sector plan" OR "government report")

**File-type & exclusion block**

filetype:(pdf OR doc OR docx)

-site:ovid.com -site:ovidsp.ovid.com -site:ebscohost.com -site:scopus.com

-site:webofscience.com -site:webofknowledge.com -site:globalindexmedicus.net

-site:lilacs.bvsalud.org -site:bvsalud.org -site:pubmed.ncbi.nlm.nih.gov

-site:sciencedirect.com -"journal article" -"systematic review"

**Example (**Running search for SAO Density for Site-Block A)

*("SAO density" OR "specialist surgical workforce density"*

*OR "surgeons anaesthesiologists obstetricians per 100000"*

*OR "surgeon density")*

**AND**

*("policy brief" OR "national surgical plan" OR NSOAP OR strategy OR roadmap*

*OR "white paper" OR "country report" OR "situation analysis"*

*OR "health sector plan" OR "government report")*

**AND**

*(site:gov.ng OR site:gov.zm OR site:go.ke OR site:gov.cv OR site:gouv.cd*

*OR site:gov.na OR site:gov.sl OR site:gov.sb OR site:gov.za OR site:go.tz*

*OR site:go.ug OR site:gov.rw OR site:gouv.sn OR site:gov.zw OR site:gov.mw*

*OR site:gov.ao OR site:gov.bw OR site:gov.cm OR site:gouv.mg OR site:gov.mz*

*OR site:gov.et OR site:gov.pk OR site:gov.ls OR site:gov.sz OR site:gov.sc*

*OR site:gov.bj)*

*filetype:(pdf OR doc OR docx)*

*-site:ovid.com -site:ovidsp.ovid.com -site:ebscohost.com -site:scopus.com*

*-site:webofscience.com -site:webofknowledge.com -site:globalindexmedicus.net*

*-site:lilacs.bvsalud.org -site:bvsalud.org -site:pubmed.ncbi.nlm.nih.gov*

*-site:sciencedirect.com -"journal article" -"systematic review"*

**Step-by-Step Search**

1. Choose indicator block – copy terms for one LCoGS indicator (see Appendix A).

2. Add policy block – paste standard policy/grey-literature term set.

3. Select site-block – run query three times (A, B, C) to cover all 48 domains.

4. Apply filters – filetype:(pdf OR doc OR docx); exclude academic sources.

5. Run search – incognito Chrome, 1 Jan 2019 → search date, results/page = 100.

6. Export results – scrape first 5 SERP pages (≤500 hits) with DataMiner.

7. Deduplicate and tag – in Excel, by indicator and site-block.

8. Screen and extract – apply inclusion criteria; record key data fields.

**Supplementary Results**

Additional details supporting the main analyses are provided in the accompanying Supplementary Tables.

These include full database search strategies (Table S1), indicator and policy-domain search blocks (Tables S2–S3), definitions and benchmarks (Table S4), GATHER checklist and quality-assessment tool (Tables S5–S6), country-level indicator data by income group (Tables S7–S11), and domain-level scoring of modelled studies (Supplementary Figure 3).

**Supplementary Appendix: PRISMA 2020 Checklist**

| **Section and Topic** | **Item #** | **Checklist item** | **Location where item is reported** |
| --- | --- | --- | --- |
| **TITLE** | | |  |
| Title | 1 | Identify the report as a systematic review. | 1 |
| **ABSTRACT** | | |  |
| Abstract | 2 | See the PRISMA 2020 for Abstracts checklist. | 2 |
| **INTRODUCTION** | | |  |
| Rationale | 3 | Describe the rationale for the review in the context of existing knowledge. | 3 |
| Objectives | 4 | Provide an explicit statement of the objective(s) or question(s) the review addresses. | 3 |
| **METHODS** | | |  |
| Eligibility criteria | 5 | Specify the inclusion and exclusion criteria for the review and how studies were grouped for the syntheses. | 4 |
| Information sources | 6 | Specify all databases, registers, websites, organisations, reference lists and other sources searched or consulted to identify studies. Specify the date when each source was last searched or consulted. | 4 |
| Search strategy | 7 | Present the full search strategies for all databases, registers and websites, including any filters and limits used. |  |
| Selection process | 8 | Specify the methods used to decide whether a study met the inclusion criteria of the review, including how many reviewers screened each record and each report retrieved, whether they worked independently, and if applicable, details of automation tools used in the process. | 5 |
| Data collection process | 9 | Specify the methods used to collect data from reports, including how many reviewers collected data from each report, whether they worked independently, any processes for obtaining or confirming data from study investigators, and if applicable, details of automation tools used in the process. | 5 |
| Data items | 10a | List and define all outcomes for which data were sought. Specify whether all results that were compatible with each outcome domain in each study were sought (e.g. for all measures, time points, analyses), and if not, the methods used to decide which results to collect. | 5 |
|  | 10b | List and define all other variables for which data were sought (e.g. participant and intervention characteristics, funding sources). Describe any assumptions made about any missing or unclear information. | 5 |
| Study risk of bias assessment | 11 | Specify the methods used to assess risk of bias in the included studies, including details of the tool(s) used, how many reviewers assessed each study and whether they worked independently, and if applicable, details of automation tools used in the process. | 5 |
| Effect measures | 12 | Specify for each outcome the effect measure(s) (e.g. risk ratio, mean difference) used in the synthesis or presentation of results. | 5 |
| Synthesis methods | 13a | Describe the processes used to decide which studies were eligible for each synthesis (e.g. tabulating the study intervention characteristics and comparing against the planned groups for each synthesis (item #5)). | 7 |
|  | 13b | Describe any methods required to prepare the data for presentation or synthesis, such as handling of missing summary statistics, or data conversions. |  |
|  | 13c | Describe any methods used to tabulate or visually display results of individual studies and syntheses. |  |
|  | 13d | Describe any methods used to synthesize results and provide a rationale for the choice(s). If meta-analysis was performed, describe the model(s), method(s) to identify the presence and extent of statistical heterogeneity, and software package(s) used. |  |
|  | 13e | Describe any methods used to explore possible causes of heterogeneity among study results (e.g. subgroup analysis, meta-regression). |  |
|  | 13f | Describe any sensitivity analyses conducted to assess robustness of the synthesized results. |  |
| Reporting bias assessment | 14 | Describe any methods used to assess risk of bias due to missing results in a synthesis (arising from reporting biases). |  |
| Certainty assessment | 15 | Describe any methods used to assess certainty (or confidence) in the body of evidence for an outcome. |  |
| **RESULTS** | | |  |
| Study selection | 16a | Describe the results of the search and selection process, from the number of records identified in the search to the number of studies included in the review, ideally using a flow diagram. | 8 |
|  | 16b | Cite studies that might appear to meet the inclusion criteria, but which were excluded, and explain why they were excluded. |  |
| Study characteristics | 17 | Cite each included study and present its characteristics. | 17 |
| Risk of bias in studies | 18 | Present assessments of risk of bias for each included study. |  |
| Results of individual studies | 19 | For all outcomes, present, for each study: (a) summary statistics for each group (where appropriate) and (b) an effect estimate and its precision (e.g. confidence/credible interval), ideally using structured tables or plots. |  |
| Results of syntheses | 20a | For each synthesis, briefly summarise the characteristics and risk of bias among contributing studies. | 19 |
|  | 20b | Present results of all statistical syntheses conducted. If meta-analysis was done, present for each the summary estimate and its precision (e.g. confidence/credible interval) and measures of statistical heterogeneity. If comparing groups, describe the direction of the effect. |  |
|  | 20c | Present results of all investigations of possible causes of heterogeneity among study results. |  |
|  | 20d | Present results of all sensitivity analyses conducted to assess the robustness of the synthesized results. |  |
| Reporting biases | 21 | Present assessments of risk of bias due to missing results (arising from reporting biases) for each synthesis assessed. |  |
| Certainty of evidence | 22 | Present assessments of certainty (or confidence) in the body of evidence for each outcome assessed. |  |
| **DISCUSSION** | | |  |
| Discussion | 23a | Provide a general interpretation of the results in the context of other evidence. | 10 |
|  | 23b | Discuss any limitations of the evidence included in the review. | 11 |
|  | 23c | Discuss any limitations of the review processes used. | 12 |
|  | 23d | Discuss implications of the results for practice, policy, and future research. | 12,13 |
| **OTHER INFORMATION** | | |  |
| Registration and protocol | 24a | Provide registration information for the review, including register name and registration number, or state that the review was not registered. | 4 |
|  | 24b | Indicate where the review protocol can be accessed, or state that a protocol was not prepared. | 4 |
|  | 24c | Describe and explain any amendments to information provided at registration or in the protocol. |  |
| Support | 25 | Describe sources of financial or non-financial support for the review, and the role of the funders or sponsors in the review. | Sup. 1 |
| Competing interests | 26 | Declare any competing interests of review authors. | Sup. 1 |
| Availability of data, code and other materials | 27 | Report which of the following are publicly available and where they can be found: template data collection forms; data extracted from included studies; data used for all analyses; analytic code; any other materials used in the review. |  |

**Supplementary Figures and Tables**

**Table S1. Bibliographic database search strategy for identification of peer-reviewed studies**

| Database | Search strategy | Result |
| --- | --- | --- |
| PUBMED | (((("lancet"[All Fields] OR "lancet s"[All Fields] OR "lancets"[All Fields]) AND ("commission"[All Fields] OR "commission s"[All Fields] OR "commissioned"[All Fields] OR "commissioning"[All Fields] OR "commissions"[All Fields]) AND ("glob surg"[Journal] OR ("global"[All Fields] AND "surgery"[All Fields]) OR "global surgery"[All Fields])) OR "LCoGS"[All Fields] OR "core surg* indicator*"[All Fields] OR "surg* indicator*"[All Fields] OR "global surg* indicator*"[All Fields] OR (("lancet"[All Fields] OR "lancets"[All Fields]) AND "surg*"[All Fields] AND "indicator*"[All Fields])) AND 2015/01/01:2024/12/31[Date - Publication]) AND (2015:2024[pdat]) | 416 |
| EMBASE via OVID | 1. "Lancet Commission on Global Surgery".mp. 2. LCoGS.mp. 3. “Core surg* indicators”.mp. 4. “Surg* Indicators”.mp. 5. "Global Surg* Indicator*".mp. 6. "Lancet surg* indicator*".mp. 7. 1 or 2 or 3 or 4 or 5 or 6 | 391 |
| ESCI via WOS | (((((ALL=("Lancet Commission on Global Surgery")) OR ALL=(LCoGS)) OR ALL=(("Core surg* indicator*"))) OR ALL=("Surg* Indicators")) OR ALL=("Global Surg* Indicator*")) OR ALL=("Lancet surg* indicator*") | 31 |
| GLobal Health (EBSCO, CABI) | AllField:("Lancet Commission on Global Surgery") OR AllField:(LCoGS) OR AllField:("Core surg* indicators") OR AllField:("Surg* Indicators") OR AllField:("Global Surg* Indicator*") OR AllField:("Lancet surg* indicator*") | 62 |
| LILACS | (("Lancet Commission on Global Surgery")) OR (lcogs) OR ((“core surg* indicators”)) OR ((“surg* indicators”) ) OR ( ("Global Surg* Indicator*")) OR ( ("Lancet surg* indicator*")) | 599 |
| WHO Global Index Medicus | (("Lancet Commission on Global Surgery")) OR (lcogs) OR ((“core surg* indicators”)) OR ((“surg* indicators”) ) OR ( ("Global Surg* Indicator*")) OR ( ("Lancet surg* indicator*")) | 46 |

**Table S2. Search terms for each Lancet Commission on Global Surgery (LCoGS) indicator (Indicator blocks)**

| **Access to timely essential surgery** | ("access to timely essential surgery" OR "2 hour surgical access" OR "two-hour access radius" OR "distance to surgery" OR "surgical travel time") |
| --- | --- |
| **Specialist surgical workforce density** | ("SAO density" OR "specialist surgical workforce density" OR "surgeons anaesthesiologists obstetricians per 100000" OR "surgeon density") |
| **Surgical volume** | ("surgical volume 5000" OR "operative procedures per 100000" OR "operations 100000" OR "surgeries per year" OR "operation rate") |
| **Perioperative Mortality Rate** | ("perioperative mortality rate" OR "postoperative mortality" OR POMR OR "surgical mortality" OR "operative deaths") |
| **Protection against impoverishing expenditure** | ("impoverishing surgical expenditure" OR "impoverishing health payments surgery" OR "pushed into poverty by surgery") |
| **Protection against catastrophic expenditure** | ("catastrophic surgical expenditure" OR "catastrophic health payments surgery" OR "financial catastrophe surgery") |

**Table S3. Country domain blocks used for Google web search (site: operators)**

| **Site-Block A (Africa-core)** | (site:gov.ng OR site:gov.zm OR site:go.ke OR site:gov.cv OR site:gouv.cd  OR site:gov.na OR site:gov.sl OR site:gov.sb OR site:gov.za OR site:go.tz  OR site:go.ug OR site:gov.rw OR site:gouv.sn OR site:gov.zw OR site:gov.mw  OR site:gov.ao OR site:gov.bw OR site:gov.cm OR site:gouv.mg OR site:gov.mz  OR site:gov.et OR site:gov.pk OR site:gov.ls OR site:gov.sz OR site:gov.sc  OR site:gov.bj) |
| --- | --- |
| **Site-Block B (Pacific + Indian Ocean)** | (site:gov.fj OR site:gouv.pf OR site:gov.my OR site:gov.mh OR site:gov.mu  OR site:gov.nr OR site:gov.np OR site:gov.pw OR site:gov.vu OR site:gov.tv  OR site:gov.to OR site:gov.fm OR site:gov.ki OR site:gov.ws OR site:gouv.wf) |
| **Site-Block C (Latin America + island overlap)** | (site:gob.ec OR site:gov.co OR site:gob.mx OR site:gov.hn OR site:gov.tt  OR site:gov.dm OR site:gov.pr OR site:gov.ag OR site:gov.bb OR site:gov.dm  OR site:gov.bs OR site:gov.lc OR site:gov.vc OR site:gov.gy) |

**Table S4. Definitions and global targets for the six Lancet Commission on Global Surgery indicators**^1^

| **Indicator** | **Definition** | **Target** |
| --- | --- | --- |
| **Group 1: Preparedness for surgical and anaesthesia care** | |  |
| **Access to timely essential surgery** | Proportion of the population that can access, within 2 h a facility that can do caesarean delivery, laparotomy, and treatment of open fracture (the Bellwether Procedures) | A minimum of 80% coverage of essential surgical and anaesthesia services per country by 2030 |
| **Specialist surgical workforce density** | Number of specialist surgical, anaesthetic, and obstetric  physicians who are working per 100 000 population | 100% of countries with at least 20 surgical, anaesthetic, and obstetric physicians per 100 000 population by 2030 |
| **Group 2: Delivery of surgical and anaesthesia care** | |  |
| **Surgical volume** | Procedures done in an operating theatre, per 100 000 population per year | 80% of countries by 2020 and 100% of countries by 2030 tracking surgical volume |
|  |  | 5000 procedures per 100,000 population by 2030 |
| **Perioperative Mortality Rate** | All-cause death rate before discharge in patients who have had a procedure in an operating theatre, divided by the total number of procedures, presented as a percentage | 80% of countries by 2020 and 100% of countries by 2030 tracking perioperative mortality |
|  |  | In 2020, assess global data and set national targets for 2030 |
| **Group 3: Effect of surgical and anaesthesia care** | |  |
| **Protection against impoverishing expenditure** | Proportion of households protected against impoverishment from direct out-of-pocket payments for surgical and anaesthesia care | 100% protection against impoverishment from out-of-pocket payments for surgical and anaesthesia care by 2030 |
| **Protection against catastrophic expenditure** | Fraction of households protected against catastrophic expenditure from direct out-of- pocket payments for surgical and anaesthesia care | 100% protection against catastrophic expenditure from out-of-pocket payments for surgical and anaesthesia care by 2030 |

**Table S5. GATHER (Guidelines for Accurate and Transparent Health Estimates Reporting) checklist items**^2^

| Item | Checklist item |
| --- | --- |
| **Objectives and funding** | |
| 1 | Define the indicator(s), populations (including age, sex, and geographic entities), and time period(s) for which estimates were made. |
| 2 | List the funding sources for the work. |
| **Data Inputs** | |
| *For all data inputs from multiple sources that are synthesized as part of the study:* | |
| 3 | Describe how the data were identified and how the data were accessed. |
| 4 | Specify the inclusion and exclusion criteria. Identify all ad‐hoc exclusions. |
| 5 | Provide information on all included data sources and their main characteristics. For each data source used, report reference information or contact name/institution, population represented, data collection method, year(s) of data collection, sex and age range, diagnostic criteria or measurement method, and sample size, as relevant. |
| 6 | Identify and describe any categories of input data that have potentially important biases (e.g., based on characteristics listed in item 5). |
| *For data inputs that contribute to the analysis but were not synthesized as part of the study:* | |
| 7 | Describe and give sources for any other data inputs. |
| **For all data inputs:** | |
| 8 | Provide all data inputs in a file format from which data can be efficiently extracted (e.g., a spreadsheet rather than a PDF), including all relevant meta‐data listed in item 5. For any data inputs that cannot be shared because of ethical or legal reasons, such as third‐party ownership, provide a contact name or the name of the institution that retains the right to the data. |
| **Data analysis** | |
| 9 | Provide a conceptual overview of the data analysis method. A diagram may be helpful. |
| 10 | Provide a detailed description of all steps of the analysis, including mathematical formulae. This description should cover, as relevant, data cleaning, data pre‐processing, data adjustments and weighting of data sources, and mathematical or statistical model(s). |
| 11 | Describe how candidate models were evaluated and how the final model(s) were selected. |
| 12 | Provide the results of an evaluation of model performance, if done, as well as the results of any relevant sensitivity analysis. |
| 13 | Describe methods for calculating uncertainty of the estimates. State which sources of uncertainty were, and were not, accounted for in the uncertainty analysis. |
| 14 | State how analytic or statistical source code used to generate estimates can be accessed. |
| **Results and Discussion** | |
| 15 | Provide published estimates in a file format from which data can be efficiently extracted |
| 16 | Report a quantitative measure of the uncertainty of the estimates (e.g. uncertainty intervals). |
| 17 | Interpret results in light of existing evidence. If updating a previous set of estimates, describe the reasons for changes in estimates. |
| 18 | Discuss limitations of the estimates. Include a discussion of any modelling assumptions or data limitations that affect interpretation of the estimates. |

**Table S6. Initial draft of the modelling study quality-assessment tool (pre-consultation version)**

| **Domains** | **Quality** | | |
| --- | --- | --- | --- |
|  | **Low** | **Intermediate** | **High** |
| **Objectives** | | | |
| 1. Provide a clear definition of the Lancet Commission indicator(s) used | No explicit definition | Definition that is slightly different to the ones used in the Lancet Commission indicators | Provides a clear definition of the indicators used, similar to the Lancet Commission Indicator |
| 2. Provide a clear definition around the population for which the modelling analysis is applicable to | No description of the population | There is a vague definition around the population (i.e., age or sex) or patients included into the study | Clear definition including what procedures are included and excluded |
| 3. Clear definition around the geography studied | No definitions of the scale of country or regions included | Clear definition around region covered but have had to model some of the data or have incomplete data for the region | Clearly defined catchment (e.g. province or state-level or national-level analysis) with complete population data |
| 4. Clear definition around the time period | Not defined | poorly defined | Clearly defined |
| **Methodology** | | | |
| 5. Description of methodology | Minimal or no description of methodology | Clearly described in text | Clearly described with conceptual overview (e.g. flowchart, diagram) and including mathematical formulae/statistical code if applicable |
| 6. Methodology of how underlying data was collected | No description on the nature of data collection for the inputs reported in the study | Data inputs obtained were retrospective in nature including administrative data, with high risk of potential biases | Data inputs were prospectively collected, either from cohort studies or randomised controlled trials |
| 7. Describe how the data inputs were identified | No description on the nature of data collection for the inputs reported in the study | Rational provided for selection of data input | Formal literature review to identify all applicable data input |
| 8. Describe how the data was accessed | No reporting of formal approval or request process | Formal request from a third-party source, with no description of the process | Formal request from a third-party source with clear description of approval process |
| 9. Specify the inclusion and exclusion criteria | No clear specification around inclusion or exclusion criteria for the data source(s) being used | Partial specification around inclusion or exclusion criteria for the data source(s) being used | Full specification around inclusion or exclusion criteria for the data source(s) being used |
| 10. Description of data source characteristics | No information on data source or characteristics | Provides information on all included data sources and their main characteristics. | Provides information on all included data sources and their main characteristics *and* identifies and describes any categories of input data that have potentially important biases |
| 11. Availability of data inputs | Not available | Data sources clearly referenced but data not provided or contacts of rights to data not provided | Provides all data inputs (including all relevant meta-data) in a file format from which data can be efficiently extracted, available in a supplementary file or contact that retains the right to the data clearly provided |
| 12. Description of statistical analysis | Minimal or no description of statistical analysis | Clearly described in text but no statistical or mathematical code provided or how they can be assessed | Detailed description of all steps of the analysis (data cleaning, data pre-processing, data adjustments and  weighting of data sources) including mathematical formulae. Mathematical or statistical model(s) available in a supplementary file |
| 13. Validation of model performance | No attempt to validate the model | Attempt to validate the underlying methodology | Attempt to validate modelled data against observed data and detailed description of how validation was done |
| 14. Sensitivity analyses | Not performed | Sensitivity analyses performed but rationale poorly explained and/or results not provided | Sensitivity analyses performed with clear explanation and results provided |
| **Interpretation** | | | |
| 15. Discussion of findings in context of existing evidence | Not performed | Relationship of findings to existing evidence identified but not explained | Relationship of findings to existing evidence identified and explained |
| 16. Identification of potential biases relating to data inputs | Not performed | Biases identified but their potential impact on findings not discussed | Biases identified and their potential impact on findings discussed |
| 17. Identification of assumptions within analytical approach | Not performed | Assumptions identified but their potential impact on findings not discussed | Assumptions identified and their potential impact on findings discussed |
| **Conflict of Interest** | | | |
| 18. Reporting of funding sources and conflicts of interest | None reported | Clear reporting of funding sources, with potential conflict since funders were involved in the analysis and interpretation of data | Clear reporting of funding sources, with no conflict since funders were not involved in the analysis and interpretation of data |

**Table S7. Countries identified in the review by World Bank income group**

| **Income group** | **Number of countries** |
| --- | --- |
| High income | 62 |
| Low income | 25 |
| Lower middle income | 50 |
| Upper middle income | 51 |
| **Total** | **188** |

**Table S8. Total World Bank countries by income group (2025 classification)**^3^

| **Income group** | **Number of countries** |
| --- | --- |
| High income | 86 |
| Low income | 26 |
| Lower middle income | 51 |
| Upper middle income | 54 |
| **Total** | **217** |

| **Tables S9. Country-level values and benchmark status for indicators 1 (Access)** | | | |
| --- | --- | --- | --- |
| **Country** | **Income Group** | **Count** | **Indicator Met** |
| Andorra | High income | 100^4^ | FALSE |
| Angola | Lower middle income | 63.1^5^ | TRUE |
| Australia | High income | 98.85^6^ | FALSE |
| Austria | High income | 100^4^ | FALSE |
| Bangladesh | Lower middle income | 50.6^7^ | TRUE |
| Belgium | High income | 100^4^ | FALSE |
| Belize | Upper middle income | 95^4^ | FALSE |
| Benin | Lower middle income | 76.7^5^ | TRUE |
| Bolivia | Lower middle income | 52^7^ | TRUE |
| Botswana | Upper middle income | 76.7^5^ | TRUE |
| Brazil | Upper middle income | 97.2^8^ | FALSE |
| Burkina Faso | Low income | 53.1^5^ | TRUE |
| Burundi | Low income | 95.7^5^ | FALSE |
| Cabo Verde | Lower middle income | 93.4^5^ | FALSE |
| Cameroon | Lower middle income | 33.1^9^ | TRUE |
| Central African Republic | Low income | 48.5^5^ | TRUE |
| China | Upper middle income | 38.6^9^ | TRUE |
| Colombia | Upper middle income | 84.9^10^ | FALSE |
| Comoros | Lower middle income | 96.6^5^ | FALSE |
| Congo, Dem. Rep | Low income | 53.7^5^ | TRUE |
| Congo, Rep. | Lower middle income | 72.3^5^ | TRUE |
| Côte d'Ivoire | Lower middle income | 65.6^5^ | TRUE |
| Cyprus | High income | 100^4^ | FALSE |
| Djibouti | Lower middle income | 83.3^5^ | FALSE |
| Equatorial Guinea | Upper middle income | 75.8^5^ | TRUE |
| Eritrea | Low income | 42.6^5^ | TRUE |
| Eswatini | Lower middle income | 93.9^5^ | FALSE |
| Ethiopia | Low income | 33.1^9^ | TRUE |
| Fiji | Upper middle income | 67^6^ | TRUE |
| Finland | High income | 99^4^ | FALSE |
| Gabon | Upper middle income | 83.6^5^ | FALSE |
| Gambia, The | Low income | 71.5^5^ | TRUE |
| Ghana | Lower middle income | 33.1^9^ | TRUE |
| Guatemala | Upper middle income | 52.4^11^ | TRUE |
| Guinea | Lower middle income | 62.7^5^ | TRUE |
| Guinea-Bissau | Low income | 61.5^5^ | TRUE |
| Iceland | High income | 94^4^ | FALSE |
| India | Lower middle income | 27.2^9^ | TRUE |
| Iran, Islamic Rep. | Upper middle income | 20.2^9^ | TRUE |
| Kenya | Lower middle income | 33.1^9^ | TRUE |
| Kiribati | Lower middle income | 65^6^ | TRUE |
| Lao PDR | Lower middle income | 56.5^4^ | TRUE |
| Latvia | High income | 100^4^ | FALSE |
| Lesotho | Lower middle income | 56.7^5^ | TRUE |
| Liberia | Low income | 61.5^12^ | TRUE |
| Lithuania | High income | 100^4^ | FALSE |
| Malaysia | Upper middle income | 94^13^ | FALSE |
| Madagascar | Low income | 29^14^ | TRUE |
| Malawi | Low income | 92.8^5^ | FALSE |
| Mali | Low income | 63.8^5^ | TRUE |
| Mauritania | Lower middle income | 38.6^5^ | TRUE |
| Mexico | Upper middle income | 81.7^15^ | FALSE |
| Monaco | High income | 100^4^ | FALSE |
| Mongolia | Upper middle income | 80.1^16^ | FALSE |
| Morocco | Lower middle income | 92^4^ | FALSE |
| Mozambique | Low income | 50.1^5^ | TRUE |
| Namibia | Upper middle income | 76.8^5^ | TRUE |
| Nauru | High income | 100^6^ | FALSE |
| Nepal | Lower middle income | 20.2^9^ | TRUE |
| Netherlands | High income | 92^4^ | FALSE |
| New Caledonia | High income | 89^1^ | FALSE |
| New Zealand | High income | 90^6^ | FALSE |
| Niger | Low income | 42.8^5^ | TRUE |
| Nigeria | Lower middle income | 33.1^9^ | TRUE |
| Pakistan | Lower middle income | 20.2^9^ | TRUE |
| Palau | High income | 76^17^ | TRUE |
| Papua New Guinea | Lower middle income | 20^6^ | TRUE |
| Philippines | Lower middle income | 20.2^18^ | TRUE |
| Rwanda | Low income | 88.8^5^ | FALSE |
| Samoa | Lower middle income | 68^6^ | TRUE |
| São Tomé and Principe | Lower middle income | 97.3^5^ | FALSE |
| Senegal | Lower middle income | 60.3^5^ | TRUE |
| Seychelles | High income | 84^4^ | FALSE |
| Sierra Leone | Low income | 60.4^5^ | TRUE |
| Solomon Islands | Lower middle income | 20^6^ | TRUE |
| Somalia | Low income | 56^5^ | TRUE |
| South Africa | Upper middle income | 33.1^9^ | TRUE |
| South Sudan | Low income | 22.8^5^ | TRUE |
| Sri Lanka | Lower middle income | 100^5^ | FALSE |
| St. Vincent and the Grenadines | Upper middle income | 100^4^ | FALSE |
| Sudan | Low income | 46.2^4^ | TRUE |
| Sweden | High income | 99^5^ | FALSE |
| Tanzania | Lower middle income | 33.1^4^ | TRUE |
| Thailand | Upper middle income | 20.2^9^ | TRUE |
| Timor-Leste | Lower middle income | 50^9^ | TRUE |
| Togo | Low income | 85.3^6^ | FALSE |
| Tonga | Upper middle income | 85^5^ | FALSE |
| Tuvalu | Upper middle income | 56^6^ | TRUE |
| Uganda | Low income | 33.1^6^ | TRUE |
| Vanuatu | Lower middle income | 44^9^ | TRUE |
| Vietnam | Lower middle income | 20.2^9^ | TRUE |
| Zambia | Lower middle income | 19.3^19^ | TRUE |
| Zimbabwe | Lower middle income | 79.3^5^ | TRUE |

**Table S10. Country-level values and benchmark status for indicator 2 (Workforce)**

| **Country** | **Income bracket** | **SAO/100 000** | **Met LCoGS Target** |
| --- | --- | --- | --- |
| Albania | Upper middle income | 6.5^20^ | TRUE |
| Algeria | Upper middle income | 12.1^4^ | TRUE |
| Andorra | High income | 55.5^4^ | FALSE |
| Antigua and Barbuda | High income | 13.1^4^ | TRUE |
| Armenia | Upper middle income | 102.4^4^ | FALSE |
| Australia | High income | 63.9^6^ | FALSE |
| Austria | High income | 110^4^ | FALSE |
| Azerbaijan | Upper middle income | 62.8^20^ | FALSE |
| Bahamas, The | High income | 28.21^21^ | FALSE |
| Bahrain | High income | 14.4^4^ | TRUE |
| Bangladesh | Lower middle income | 3^4^ | TRUE |
| Barbados | Upper middle income | 17.3^4^ | TRUE |
| Belarus | Upper middle income | 121.3^4^ | FALSE |
| Belgium | High income | 78.2^4^ | FALSE |
| Belize | Upper middle income | 10.5^4^ | TRUE |
| Benin | Lower middle income | 1.9^4^ | TRUE |
| Bhutan | Lower middle income | 2.8^4^ | TRUE |
| Bolivia | Lower middle income | 34.9^4^ | FALSE |
| Bosnia and Herzegovina | Upper middle income | 26.3^4^ | FALSE |
| Botswana | Upper middle income | 3.5^4^ | TRUE |
| Brazil | Upper middle income | 34.7^22^ | FALSE |
| Bulgaria | High income | 120.8^4^ | FALSE |
| Burkina Faso | Low income | 0.6^4^ | TRUE |
| Cabo Verde | Lower middle income | 15.7^4^ | TRUE |
| Cambodia | Lower middle income | 4.2^3^ | TRUE |
| Cameroon | Lower middle income | 1.7^4^ | TRUE |
| Canada | High income | 36.1^4^ | FALSE |
| Central African Republic | Low income | 0.4^4^ | TRUE |
| Chad | Low income | 0.3^4^ | TRUE |
| Chile | High income | 59.6^4^ | FALSE |
| China | Upper middle income | 40.1^4^ | FALSE |
| Colombia | Upper middle income | 11·9^10^ | FALSE |
| Congo, Dem. Rep | Low income | 0.18^22^ | TRUE |
| Congo, Rep. | Lower middle income | 0.2^4^ | TRUE |
| Costa Rica | Upper middle income | 22.3^4^ | FALSE |
| Côte d'Ivoire | Lower middle income | 2.8^4^ | TRUE |
| Croatia | High income | 81.2^4^ | FALSE |
| Cuba | Upper middle income | 90.1^4^ | FALSE |
| Cyprus | High income | 83.3^4^ | FALSE |
| Czechia | High income | 114.9^4^ | FALSE |
| Denmark | High income | 69^4^ | FALSE |
| Djibouti | Lower middle income | 4.3^4^ | TRUE |
| Dominica | Upper middle income | 11^4^ | TRUE |
| Ecuador | Upper middle income | 59.6^4^ | FALSE |
| Egypt, Arab Rep. | Lower middle income | 48.9^4^ | FALSE |
| Estonia | High income | 94.3^4^ | FALSE |
| Ethiopia | Low income | 0.6^4^ | TRUE |
| Fiji | Upper middle income | 5.8^6^ | TRUE |
| Finland | High income | 110.1^4^ | FALSE |
| France | High income | 54.7^4^ | FALSE |
| French Polynesia | High income | 32.6^3^ | FALSE |
| Gambia, The | Low income | 1.1^4^ | TRUE |
| Georgia | Upper middle income | 73.7^20^ | FALSE |
| Germany | High income | 108^4^ | FALSE |
| Greece | High income | 163.5^4^ | FALSE |
| Grenada | Upper middle income | 11.3^4^ | TRUE |
| Guatemala | Upper middle income | 3.4^4^ | TRUE |
| Guyana | High income | 8.8^4^ | TRUE |
| Haiti | Lower middle income | 4.5^4^ | TRUE |
| Honduras | Lower middle income | 12.4^4^ | TRUE |
| Hong Kong SAR (China) | High income | 27.8^3^ | FALSE |
| Hungary | High income | 56.7^20^ | FALSE |
| Iceland | High income | 78.9^4^ | FALSE |
| India | Lower middle income | 6.5^20^ | TRUE |
| Iran, Islamic Rep. | Upper middle income | 5^4^ | TRUE |
| Iraq | Upper middle income | 7.5^4^ | TRUE |
| Ireland | High income | 40.81^21^ | FALSE |
| Israel | High income | 68.91^21^ | FALSE |
| Italy | High income | 109.9^4^ | FALSE |
| Jamaica | Upper middle income | 11.3^4^ | TRUE |
| Japan | High income | 31.3^4^ | FALSE |
| Kazakhstan | Upper middle income | 73.1^4^ | FALSE |
| Kenya | Lower middle income | 2.65^21^ | TRUE |
| Kiribati | Lower middle income | 8.2^6^ | TRUE |
| Korea, Rep. | High income | 49.3^4^ | FALSE |
| Kyrgyz Republic | Lower middle income | 60.4^4^ | FALSE |
| Lao PDR | Lower middle income | 2.9^4^ | TRUE |
| Latvia | High income | 89.5^4^ | FALSE |
| Lebanon | Lower middle income | 70.9^4^ | FALSE |
| Lesotho | Lower middle income | 0.6^4^ | TRUE |
| Libya | Upper middle income | 0·7 (0·2-1·9) ^22^ | TRUE |
| Lithuania | High income | 111.2^4^ | FALSE |
| Luxembourg | High income | 80.8^4^ | FALSE |
| Madagascar | Low income | 0.78^14^ | TRUE |
| Malawi | Low income | 0.5^4^ | TRUE |
| Malaysia | Upper middle income | 15.58^21^ | TRUE |
| Maldives | Upper middle income | 15.96^21^ | TRUE |
| Malta | High income | 85.3^4^ | FALSE |
| Marshall Islands | Upper middle income | 17.1^4^ | TRUE |
| Mauritius | Upper middle income | 34.57^21^ | FALSE |
| Mexico | Upper middle income | 40.6^15^ | FALSE |
| Micronesia, Fed. Sts. | Lower middle income | 7^6^ | TRUE |
| Moldova | Upper middle income | 49.4^3^ | FALSE |
| Monaco | High income | 191.4^4^ | FALSE |
| Mongolia | Upper middle income | 47.4^16^ | FALSE |
| Montenegro | Upper middle income | 64.1^4^ | FALSE |
| Morocco | Lower middle income | 3.6^4^ | TRUE |
| Mozambique | Low income | 1^4^ | TRUE |
| Myanmar | Lower middle income | 2.4^4^ | TRUE |
| Namibia | Upper middle income | 5.6^4^ | TRUE |
| Nauru | High income | 30^6^ | FALSE |
| Nepal | Lower middle income | 2.8^4^ | TRUE |
| Netherlands | High income | 49^4^ | FALSE |
| New Zealand | High income | 43^6^ | FALSE |
| Nicaragua | Lower middle income | 15.8^4^ | TRUE |
| Niger | Low income | 0.4^4^ | TRUE |
| Nigeria | Lower middle income | 1.64^21^ | TRUE |
| North Macedonia | Upper middle income | 61.2^3^ | FALSE |
| Northern Mariana Islands | High income | 1.5^23^ | TRUE |
| Norway | High income | 66^4^ | FALSE |
| Oman | High income | 27.3^4^ | FALSE |
| Pakistan | Lower middle income | 6^21^ | TRUE |
| Palau | High income | 14.5^4^ | TRUE |
| Panama | High income | 26.2^4^ | FALSE |
| Papua New Guinea | Lower middle income | 2.3^6^ | TRUE |
| Paraguay | Upper middle income | 14.4^4^ | TRUE |
| Peru | Upper middle income | 41.77^21^ | FALSE |
| Philippines | Lower middle income | 9.5^4^ | TRUE |
| Poland | High income | 67.9^4^ | FALSE |
| Portugal | High income | 86.1^4^ | FALSE |
| Qatar | High income | 14.1^4^ | TRUE |
| Romania | High income | 60.5^4^ | FALSE |
| Russian Federation | High income | 63.1^4^ | FALSE |
| Rwanda | Low income | 1.69^21^ | TRUE |
| Samoa | Lower middle income | 1.6^6^ | TRUE |
| San Marino | High income | 110.2^4^ | FALSE |
| Senegal | Lower middle income | 1^4^ | TRUE |
| Serbia | Upper middle income | 64.5^4^ | FALSE |
| Seychelles | High income | 35^4^ | FALSE |
| Sierra Leone | Low income | 0.23^21^ | TRUE |
| Singapore | High income | 41.22^21^ | FALSE |
| Slovak Republic | High income | 57.2^4^ | FALSE |
| Slovenia | High income | 66.8^4^ | FALSE |
| Solomon Islands | Lower middle income | 2.5^6^ | TRUE |
| Somalia | Low income | 0.2^4^ | TRUE |
| South Africa | Upper middle income | 10.11^21^ | TRUE |
| South Sudan | Low income | 0.3^4^ | TRUE |
| Spain | High income | 87.7^4^ | FALSE |
| Sri Lanka | Lower middle income | 1.9^4^ | TRUE |
| St. Kitts and Nevis | High income | 31.6^4^ | FALSE |
| St. Lucia | Upper middle income | 23.8^4^ | FALSE |
| St. Vincent and the Grenadines | Upper middle income | 12.8^4^ | TRUE |
| Sudan | Low income | 2.8^4^ | TRUE |
| Sweden | High income | 113.1^4^ | FALSE |
| Switzerland | High income | 101^4^ | FALSE |
| Tajikistan | Lower middle income | 41.1^4^ | FALSE |
| Tanzania | Lower middle income | 0·7 (0·2-1·9) ^22^ | TRUE |
| Thailand | Upper middle income | 13.1^4^ | TRUE |
| Timor-Leste | Lower middle income | 0.9^6^ | TRUE |
| Togo | Low income | 0.5^4^ | TRUE |
| Tonga | Upper middle income | 14^6^ | TRUE |
| Trinidad and Tobago | High income | 39.64^21^ | FALSE |
| Tunisia | Lower middle income | 11.7^20^ | TRUE |
| Türkiye | Upper middle income | 47.8^4^ | FALSE |
| Turkmenistan | Upper middle income | 55.3^4^ | FALSE |
| Tuvalu | Upper middle income | 18.5^6^ | TRUE |
| Uganda | Low income | 1.57^21^ | TRUE |
| Ukraine | Upper middle income | 100.2^20^ | FALSE |
| United Kingdom | High income | 77.7^4^ | FALSE |
| United States | High income | 54.8^4^ | FALSE |
| Uruguay | High income | 38.9^4^ | FALSE |
| Uzbekistan | Lower middle income | 45.5^20^ | FALSE |
| Vanuatu | Lower middle income | 3.2^6^ | TRUE |
| Yemen, Rep. | Low income | 0.8^4^ | TRUE |
| Zambia | Lower middle income | 1.1^4^ | TRUE |
| Zimbabwe | Lower middle income | 2.48^21^ | TRUE |
| Liberia | Low income | 1.25^24^ | TRUE |

**Table S11.** **Country-level values and benchmark status for indicators 3 (Workforce)**

| **Country** | **Income group** | **SV** | **<5000** |
| --- | --- | --- | --- |
| Afghanistan | Low income | 229^25^ | TRUE |
| Albania | Upper middle income | 1,901^4^ | TRUE |
| Algeria | Upper middle income | 212 (IQR 65–578)^22^ | TRUE |
| Andorra | High income | 5,238^4^ | FALSE |
| Armenia | Upper middle income | 4,343^4^ | TRUE |
| Australia | High income | 10,156^6^ | FALSE |
| Austria | High income | 13,977^4^ | FALSE |
| Azerbaijan | Upper middle income | 1,865^4^ | TRUE |
| Bahrain | High income | 3,999^4^ | TRUE |
| Bangladesh | Lower middle income | 162^25^ | TRUE |
| Belarus | Upper middle income | 18,440^4^ | FALSE |
| Belgium | High income | 21,009^4^ | FALSE |
| Belize | Upper middle income | 1,964^4^ | TRUE |
| Benin | Lower middle income | 212 (IQR 65–578)^22^ | TRUE |
| Bhutan | Lower middle income | 2,597^4^ | TRUE |
| Bolivia | Lower middle income | 2,305^4^ | TRUE |
| Brazil | Upper middle income | 4433^8^ | TRUE |
| Bulgaria | High income | 6,884^4^ | FALSE |
| Burkina Faso | Low income | 328^4^ | TRUE |
| Burundi | Low income | 212 (IQR 65–578)^22^ | TRUE |
| Cameroon | Lower middle income | 212 (IQR 65–578)^22^ | TRUE |
| Canada | High income | 6,857^25^ | FALSE |
| Chad | Low income | 52^4^ | TRUE |
| China | Upper middle income | 2,732^4^ | FALSE |
| Colombia | Upper middle income | 2450^10^ | TRUE |
| Congo, Dem. Rep | Low income | 212 (IQR 65–578) ^22^ | TRUE |
| Congo, Rep. | Lower middle income | 212 (IQR 65–578) ^22^ | TRUE |
| Costa Rica | Upper middle income | 3,746^4^ | TRUE |
| Cuba | Upper middle income | 4,740^4^ | TRUE |
| Cyprus | High income | 3,060^4^ | TRUE |
| Czechia | High income | 6,268^4^ | FALSE |
| Denmark | High income | 10,169^4^ | FALSE |
| Ecuador | Upper middle income | 1,523^4^ | TRUE |
| Egypt, Arab Rep. | Lower middle income | 212 (IQR 65–578) ^22^ | TRUE |
| El Salvador | Upper middle income | 2,797^25^ | TRUE |
| Estonia | High income | 9,709^4^ | FALSE |
| Ethiopia | Low income | 30 to 712^26^ | TRUE |
| Fiji | Upper middle income | 1490^17^ | TRUE |
| Finland | High income | 11,592^4^ | FALSE |
| France | High income | 16,306^25^ | FALSE |
| Gambia, The | Low income | 212 (IQR 65–578) ^22^ | TRUE |
| Georgia | Upper middle income | 4,954^4^ | TRUE |
| Germany | High income | 12,188^25^ | FALSE |
| Ghana | Lower middle income | 869^27^ | TRUE |
| Guatemala | Upper middle income | 1,515^4^ | TRUE |
| Hungary | High income | 13,827^4^ | FALSE |
| India | Lower middle income | 1385.28^28^ | TRUE |
| Ireland | High income | 3,053^4^ | TRUE |
| Israel | High income | 5,067^4^ | FALSE |
| Italy | High income | 6,918^25^ | FALSE |
| Japan | High income | 1,231^29^ | TRUE |
| Jordan | Lower middle income | 2,473^4^ | TRUE |
| Kazakhstan | Upper middle income | 4,459^4^ | TRUE |
| Kenya | Lower middle income | 212 (IQR 65–578) ^22^ | TRUE |
| Kiribati | Lower middle income | 1718^6^ | TRUE |
| Korea, Rep. | High income | 3,406^4^ | TRUE |
| Kyrgyz Republic | Lower middle income | 3,045^4^ | TRUE |
| Latvia | High income | 18,404^4^ | FALSE |
| Liberia | Low income | 331^25^ | TRUE |
| Libya | Upper middle income | 212 (IQR 65–578) ^22^ | TRUE |
| Lithuania | High income | 10,189^4^ | FALSE |
| Luxembourg | High income | 21,933^25^ | FALSE |
| Madagascar | Low income | 163^14^ | TRUE |
| Mali | Low income | 212 (IQR 65–578) ^22^ | TRUE |
| Malta | High income | 13,232^4^ | FALSE |
| Mauritius | Upper middle income | 2,057^4^ | TRUE |
| Mexico | Upper middle income | 726.9^15^ | TRUE |
| Micronesia, Fed. Sts. | Lower middle income | <5000^6^ | TRUE |
| Moldova | Upper middle income | 4,898^4^ | TRUE |
| Mongolia | Upper middle income | 5784^16^ | FALSE |
| Morocco | Lower middle income | 746^4^ | TRUE |
| Myanmar | Lower middle income | 668^4^ | TRUE |
| Namibia | Upper middle income | 212 (IQR 65–578) ^22^ | TRUE |
| Nauru | High income | 7130^6^ | FALSE |
| Nepal | Lower middle income | 208^4^ | TRUE |
| Netherlands | High income | 16,639^25^ | FALSE |
| New Zealand | High income | 5308^6^ | FALSE |
| Nicaragua | Lower middle income | 4,860^4^ | TRUE |
| Niger | Low income | 255^4^ | TRUE |
| Nigeria | Lower middle income | 212 (IQR 65–578) ^22^ | TRUE |
| Oman | High income | 2,452^4^ | TRUE |
| PALAU | High income | 8606^17^ | FALSE |
| Papua New Guinea | Lower middle income | 1264^6^ | TRUE |
| Peru | Upper middle income | 3,005^4^ | TRUE |
| Poland | High income | 1,515^25^ | TRUE |
| Portugal | High income | 8,439^4^ | FALSE |
| Qatar | High income | 1,891^25^ | TRUE |
| Russian Federation | High income | 6,622^4^ | FALSE |
| Rwanda | Low income | 850^25^ | TRUE |
| Samoa | Lower middle income | 1552^6^ | TRUE |
| San Marino | High income | 4,961^4^ | TRUE |
| Saudi Arabia | High income | 3,447^4^ | TRUE |
| Senegal | Lower middle income | 212 (IQR 65–578) ^22^ | TRUE |
| Seychelles | High income | 27,351^4^ | FALSE |
| Sierra Leone | Low income | 357^4^ | TRUE |
| Slovak Republic | High income | 8,786^4^ | FALSE |
| Slovenia | High income | 7,579^4^ | FALSE |
| Solomon Islands | Lower middle income | 868^6^ | TRUE |
| Somaliland | Low income | 368^30^ | TRUE |
| South Africa | Upper middle income | 212 (IQR 65–578) ^22^ | TRUE |
| South Sudan | Low income | 262^4^ | TRUE |
| Spain | High income | 10,000^4^ | FALSE |
| Sri Lanka | Lower middle income | 1,699^4^ | TRUE |
| Sweden | High income | 15,228^4^ | FALSE |
| Switzerland | High income | 25,923^25^ | FALSE |
| Syrian Arab Republic | Low income | 1,617^4^ | TRUE |
| Tajikistan | Lower middle income | 1,678^4^ | TRUE |
| Tanzania | Lower middle income | 212 (IQR 65–578) ^22^ | TRUE |
| Timor-Leste | Lower middle income | 433^6^ | TRUE |
| Togo | Low income | 212 (IQR 65–578) ^22^ | TRUE |
| Tonga | Upper middle income | 2864^6^ | TRUE |
| Türkiye | Upper middle income | 11,911^4^ | FALSE |
| Turkmenistan | Upper middle income | 2,605^4^ | TRUE |
| Tuvalu | Upper middle income | 3417^6^ | TRUE |
| Uganda | Low income | 212 (IQR 65–578) ^22^ | TRUE |
| Ukraine | Upper middle income | 5,337^4^ | FALSE |
| United Kingdom | High income | 7,181^4^ | FALSE |
| United States | High income | 12,087^25^ | FALSE |
| Uzbekistan | Lower middle income | 2,584^4^ | TRUE |
| Vanuatu | Lower middle income | 1277^6^ | TRUE |
| Yemen, Rep. | Low income | 157^4^ | TRUE |
| Zambia | Lower middle income | 680^4^ | TRUE |
| Zimbabwe | Lower middle income | 212 (IQR 65–578) ^22^ | TRUE |

**References**

1. Meara JG, Leather AJM, Hagander L, Alkire BC, Alonso N, Ameh EA, et al. Global Surgery 2030: evidence and solutions for achieving health, welfare, and economic development. The Lancet. 2015 Aug 8;386(9993):569–624.

2. Stevens GA, Alkema L, Black RE, Boerma JT, Collins GS, Ezzati M, et al. Guidelines for Accurate and Transparent Health Estimates Reporting: the GATHER statement. Lancet. 2016 Dec 10;388(10062):e19–23.

3. World Bank Open Data. World Bank Open Data https://data. worldbank.org/indicator?tab=all. World Bank Open Data. World Bank Open Data. 2025.

4. Holmer H, Bekele A, Hagander L, Harrison EM, Kamali P, Ng-Kamstra JS, et al. Evaluating the collection, comparability and findings of six global surgery indicators. Br J Surg. 2019;106(2):e138–50.

5. Ouma PO, Maina J, Thuranira PN, Macharia PM, Alegana VA, English M, et al. Access to emergency hospital care provided by the public sector in sub-Saharan Africa in 2015: a geocoded inventory and spatial analysis. Lancet Glob Health. 2018 Mar;6(3):e342–50.

6. Guest GD, McLeod E, Perry WRG, Tangi V, Pedro J, Ponifasio P, et al. Collecting data for global surgical indicators: a collaborative approach in the Pacific region. BMJ Global Health. 2017;2(4):e000376.

7. Knowlton LM, Banguti P, Chackungal S, Chanthasiri T, Chao TE, Dahn B, et al. A geospatial evaluation of timely access to surgical care in seven countries. Bull World Health Organ. 2017 June 1;95(6):437–44.

8. Massenburg BB, Saluja S, Jenny HE, Raykar NP, Ng-Kamstra J, Guilloux AGA, et al. Assessing the Brazilian surgical system with six surgical indicators: a descriptive and modelling study. BMJ Global Health. 2017;2(2):e000226.

9. Pouramin P, Li CS, Busse JW, Sprague S, Devereaux PJ, Jagnoor J, et al. Delays in hospital admissions in patients with fractures across 18 low-income and middle-income countries (INORMUS): a prospective observational study. Lancet Global Health. 2020;8(5):e711–20.

10. Hanna JS, Herrera-Almario GE, Pinilla-Roncancio M, Tulloch D, Valencia SA, Sabatino ME, et al. Use of the six core surgical indicators from the Lancet commission on global surgery in Colombia: a situational analysis. Lancet Global Health. 2020;8(5):e699–710.

11. Buda AM, Truche P, Izquierdo E, Izquierdo S, Asturias S, Stankey M, et al. Use of geospatial analysis for priority setting in surgical system investment in Guatemala. Lancet Reg Health Am. 2022 Mar;7:100145.

12. Adde HA, van Duinen AJ, Andrews BC, Bakker J, Goyah KS, Salvesen Ø, et al. Mapping population access to essential surgical care in Liberia using equipment, personnel, and bellwether capability standards. Br J Surg. 2023 Jan 10;110(2):169–76.

13. Mapping timely access to emergency and essential surgical services: The Malaysian experience - Hoh - 2022 - ANZ Journal of Surgery - Wiley Online Library [Internet]. [cited 2025 July 11]. Available from: https://onlinelibrary.wiley.com/doi/abs/10.1111/ans.16986

14. Bruno E, White MC, Baxter LS, Ravelojaona VA, Rakotoarison HN, Andriamanjato HH, et al. An Evaluation of Preparedness, Delivery and Impact of Surgical and Anesthesia Care in Madagascar: A Framework for a National Surgical Plan. World J Surg. 2017;41(5):1218–24.

15. Pérez-Soto RH, Trolle-Silva AM, Buerba-Romero Valdés GA, Sánchez-Morales GE, Velázquez-Fernández D, Ramos-De la Medina A, et al. Timely access to essential surgery, surgical workforce, and surgical volume: global surgery indicators in Mexico. Global Health: Science and Practice. 2023;11(1).

16. Nunez JM, Nellermoe J, Davis A, Ruhnke S, Gonchigjav B, Bat-Erdene N, et al. Establishing a baseline for surgical care in Mongolia: a situational analysis using the six indicators from the Lancet Commission on Global Surgery. BMJ Open. 2022;12(7).

17. Qin RX, Zhang G, Lim MX, Waqainabete I, Tudravu J, Turagava J, et al. Assessment of essential surgical and anaesthesia care capacity: a cross-sectional study in five Pacific Island Countries. The Lancet Regional Health – Western Pacific [Internet]. 2023 Oct 1 [cited 2025 July 11];39. Available from: https://www.thelancet.com/journals/lanwpc/article/PIIS2666-6065(23)00148-7/fulltext

18. Lim X, Ayyappan M, Zaw MWW, Mandyam NK, Chia HX, Lucero-Prisno DE. Geospatial mapping of 2-hour access to timely essential surgery in the Philippines. BMJ Open. 2023 Dec;13(12):e074521.

19. Esquivel MM, Uribe-Leitz T, Makasa E, Bowman K, Weiser TG. Mapping disparities in access to surgical care: An application of geographic information systems to evaluate surgical infrastructure in Zambia. Annals of Global Health. 2016;82(3):415–6.

20. Holmer H, Lantz A, Kunjumen T, Finlayson S, Hoyler M, Siyam A, et al. Global distribution of surgeons, anaesthesiologists, and obstetricians. Lancet Glob Health. 2015 Apr 27;3 Suppl 2:S9-11.

21. Bouchard ME, Justiniano J, Vervoort D, Gore-Booth J, Emmanuel A, Langer M. Cross-sectional analysis tracking workforce density in surgery, anesthesia, and obstetrics as an indicator of progress toward improved global surgical access. IJS Global Health. 2020;3(6):e26.

22. Biccard BM, Madiba TE, Kluyts HL, Munlemvo DM, Madzimbamuto FD, Basenero A, et al. Perioperative patient outcomes in the African Surgical Outcomes Study: a 7-day prospective observational cohort study. Lancet. 2018 Apr 21;391(10130):1589–98.

23. Commonwealth Healthcare Corporation, Commonwealth of Northern Mariana Islands, 2020 [Internet]. Available from: https://www.ruralhealthinfo.org/states/northern-mariana

24. Odinkemelu DS, Sonah AK, Nsereko ET, Dahn BT, Martin MH, Moon TD, et al. An Assessment of Anesthesia Capacity in Liberia: Opportunities for Rebuilding Post-Ebola. Anesth Analg. 2021 June 1;132(6):1727–37.

25. Weiser TG, Haynes AB, Molina G, Lipsitz SR, Esquivel MM, Uribe-Leitz T, et al. Size and distribution of the global volume of surgery in 2012. Bull World Health Organ. 2016 Mar 1;94(3):201–209f.

26. Cook KR, Zeleke ZB, Gebrehana E, Burssa D, Yeshanew B, Michael A, et al. Quality and sustainability of Ethiopia’s national surgical indicators. PLOS Glob Public Health. 2024;4(3):e0002600.

27. Gyedu A, Stewart B, Gaskill C, Boakye G, Appiah-Denkyira E, Donkor P, et al. Improving benchmarks for global surgery: nationwide enumeration of operations performed in Ghana. Annals of Surgery. 2018;268(2):282–8.

28. Zadey S. Population-Level Surgical Rates in India: A Repeated Cross-Section Analysis of 737 Districts. Journal of the American College of Surgeons. 2023;237(5 Supplement 1):S231–2.

29. Action Framework for Safe and Affordable Surgery in the Western Pacific Region (2021–2030) [Internet]. Available from: https://www.who.int/docs/default-source/wpro---documents/regional-committee/session-71/rc71-7-safe-and-affordable-surgery-annex.pdf

30. Dahir S, Cotache-Condor CF, Concepcion T, Mohamed M, Poenaru D, Ismail EA, et al. Interpreting the lancet surgical indicators in Somaliland: a cross-sectional study. BMJ Open. 2020;10(12).
